# Epitranscriptomic Approach: To Improve the Efficacy of ICB Therapy by Co-Targeting Intracellular Checkpoint CISH

**DOI:** 10.3390/cells10092250

**Published:** 2021-08-30

**Authors:** Sunil Kumar, Parth Sarthi, Indra Mani, Muhammad Umer Ashraf, Myeong-Ho Kang, Vishal Kumar, Yong-Soo Bae

**Affiliations:** 1 Department of Biological Sciences, Sungkyunkwan University, Jangan-gu, Suwon 16419, Gyeonggi-do, Korea; drumerashraf@gmail.com (M.U.A.); mho231@nate.com (M.-H.K.); 2 Science Research Center (SRC) for Immune Research on Non-lymphoid Organ (CIRNO), Sungkyunkwan University, Jangan-gu, Suwon 16419, Gyeonggi-do, Korea; 3 University Department of Botany, M.Sc. Biotechnology, Ranchi University, Ranchi 834008, India; Parthneet@gmail.com; 4 Department of Microbiology, Gargi College, University of Delhi, New Delhi 110049, India; indramanibhu@gmail.com; 5 Department of Pharmaceutical Science, Dayananda Sagar University, Bengaluru 560078, India; vk861406@gmail.com

**Keywords:** epitranscriptomics, immune checkpoint blockage (ICB) therapy, anti-PD-1/PD-L1 drug resistance, personalized medicine, CISH, microRNAs

## Abstract

Cellular immunotherapy has recently emerged as a fourth pillar in cancer treatment co-joining surgery, chemotherapy and radiotherapy. Where, the discovery of immune checkpoint blockage or inhibition (ICB/ICI), anti-PD-1/PD-L1 and anti-CTLA4-based, therapy has revolutionized the class of cancer treatment at a different level. However, some cancer patients escape this immune surveillance mechanism and become resistant to ICB-therapy. Therefore, a more advanced or an alternative treatment is required urgently. Despite the functional importance of epitranscriptomics in diverse clinico-biological practices, its role in improving the efficacy of ICB therapeutics has been limited. Consequently, our study encapsulates the evidence, as a possible strategy, to improve the efficacy of ICB-therapy by co-targeting molecular checkpoints especially N^6^A-modification machineries which can be reformed into RNA modifying drugs (RMD). Here, we have explained the mechanism of individual RNA-modifiers (editor/writer, eraser/remover, and effector/reader) in overcoming the issues associated with high-dose antibody toxicities and drug-resistance. Moreover, we have shed light on the importance of suppressor of cytokine signaling (SOCS/CISH) and microRNAs in improving the efficacy of ICB-therapy, with brief insight on the current monoclonal antibodies undergoing clinical trials or already approved against several solid tumor and metastatic cancers. We anticipate our investigation will encourage researchers and clinicians to further strengthen the efficacy of ICB-therapeutics by considering the importance of epitranscriptomics as a personalized medicine.

## 1. Introduction 

**Figure 1:**
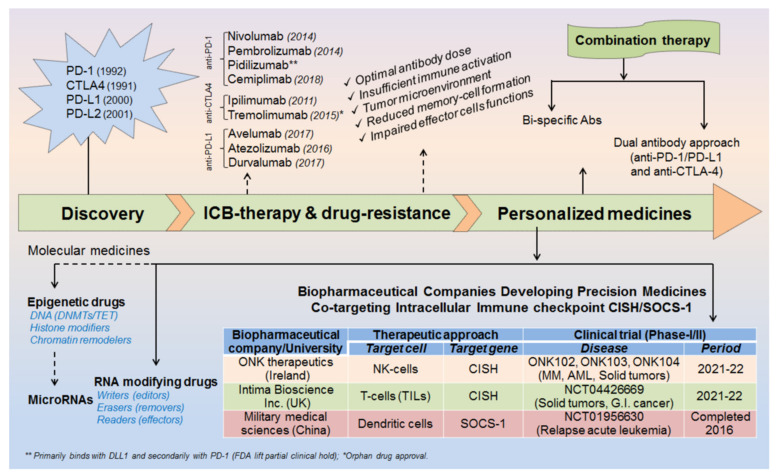
Milestones in the development of ICB-therapeutics. Discovery of immune checkpoint markers. Year of FDA-approved ICB-antibodies. Factor affecting antibody/drug-resistance. Recent strategies to improve ICB-efficacy by combining molecular medicines. Biopharmaceutical companies developing personalized medicines co-targeting epitranscriptomics and intracellular immune checkpoint (CISH/SOCS-1) in NK-cells, TILs and DCs with relevant clinical trial were summarized.

The advent of immunotherapy given in-combination with standard chemotherapeutic drugs has greatly control the cancer-spread over decades. However, still some cancer patients develop resistance against these therapeutic approaches, alarming the discovery of further advanced medicines. The invention of programmed cell death protein-1 and its ligand-1 (PD-1/PD-L1) was breakthrough in the history of cancer treatment, but still some tumors escape these immune surveillance mechanisms and relapse to grow continuously. Therefore, a more creative and advanced treatment is required instantly to overcome the issues largely associated with high-dose antibody/drug toxicities and drug-resistances, conceivably in the form of personalized medicines. In this study, we have summarized epitranscriptomic mechanisms to improve the efficacy of ICB-therapy by targeting N^6^A-modification machineries especially m^6^A-modifiers. Moreover, we have also emphasized co-targeting immune checkpoint proteins (PD-1 and PD-L1) along with intracellular checkpoint molecules (CISH, SOCS-1 and microRNAs) in enhancing the efficacy of ICB-therapeutics by combining immunotherapy. A recent human clinical trial NCT04426669, NCT03538613 [1,2] evidenced the success of targeting CISH/SOCS-1 in NK-cells [3,4], T-cells [5,6], and DCs [7] in further strengthening the efficacy of ICB-therapy against broad range of solid tumors and metastatic gastrointestinal cancers (Table 1, Table 2 and Table 3) and (Figure 1).

**Figure 2:**
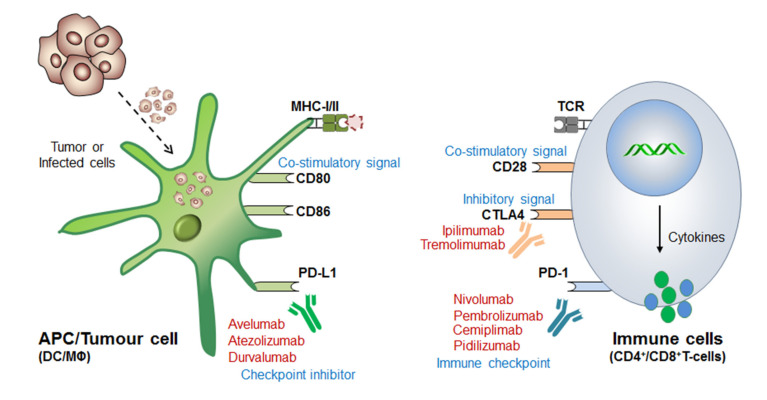
Mechanism of immune checkpoint blockage or inhibition (ICB/ICI) therapy. The antigen presenting cells (APCs), especially dendritic cells and macrophages recognize and engulf the virus-infected or cancerous cells. The immune cells now processed and present the antigen to the naive T-cells in conjugation with MHC-I/II. The T-cell receptor (TCR) present on the immune cells recognizes this processed antigen and activates humoral as well as cell-mediated immune response. However, interestingly, immune cells, like CD8^+^T-cells also express PD-1 marker which function as “immune checkpoint” before cytolytic activation. On the other hand, tumor-engulfed DCs also expresses PD-L1 and PD-L2 (ligand for PD-1) and inhibitor bypass the function of immune activation called “immune checkpoint inhibitor” and thus T-cells filed to recognize it and considered as ‘self’ rather than ‘foreign’. Therefore tumor cell escapes this immune-surveillance mechanism and proliferates rapidly. Blocking these immune checkpoint markers by means of specific antibodies endorsed the discovery of ICB-therapeutics, for example, (i) Anti-PD-1 therapy (or Immune cell targeted therapy): Nivolumab (Opdivo®), Pembrolizumab (Keytruda®), Pedilizumab (CT-011) and Cemiplimab (Libtayo®) block PD-1 receptor and bypass the ‘self-recognition’ mechanism of T-cells, and thereby allowing rapid recognition and cytolytic activation to kill tumor cells. (ii) Anti-CTLA4 therapy: Immune cell (T-cells) expresses CTLA-4 to maintain normal homeostasis by regulating the hyper activation of other immune cells and also to avoid autoimmunity, just like ‘speed breaker’. But due to its impairments under the TME it is required to be constantly activated, and so anti-CTLA4 antibodies, like Ipilimumab (Yervoy®) and Tremolimumab efficiently block the inhibitory effect of CTLA-4. Moreover, since it is highly homologous to CD28-receptor functions, thereby further activating CD8^+^T effector function to enhance anti-tumor immunity. (iii) Tumor targeted therapy (or, immune checkpoint inhibitor): The anti-PD-L1 antibodies, like Atezolizumab (Tecentriq®), Avelumab (Bavencio®) and Durvalumab (Imfinzi®) blocks the inhibitory signal generated by tumor expressing PD-L1 (ligand for PD-1) to stop its self-defense mechanism, resulting in rapid tumor killing by T-cell attack. The detail mechanism of antigen presentation, ICB-therapy and strategies to overcome drug-resistance is well discribed in these articles [64,75].

Before coming to the main stream of this review, a very logical question arises: (i) why even after so strong therapeutic approaches still some cancer cells escape these immune surveillance mechanisms? (ii) What could be the best possible combinations to overcome the issues associated with drug-resistance and high-dose antibody toxicities [72,73,74,75] and (iii) what would be the best diagnostic biomarkers or alternative strategies to completely eliminate these cancerous cells? Such questions provoked the scientist to further understand in-depth of the molecular mechanism of immune cell regulation and ICB-drug resistance. This revealed that; immune cells contains both inhibitory (break) as well as activator (acceleratory) marker to maintain immune homeostasis, or to avoid a situation called autoimmunity and self-tolerance phenomena. This understanding led to the discovery of (i) first immune checkpoint marker PD-1 or PDCD1 (CD279) in 1992 [76] and (ii) immune cell inhibitory marker cytotoxic T-lymphocyte-associated protein-4 (CTLA-4 or CD152) in 1991 [77] or 1995 [78,79]. However the first anti-CTLA4-based therapy ‘Ipilimumab’ was approved in 2011 by (James P. Allison, Nobel laureate, physiology or medicine, 2018) Medarex and Bristol-Myers Squibb for the treatment of melanoma, and the first anti-PD-1 therapy was approved in 2014 for melanoma and in 2015 for non-small-cell lung carcinoma (NSCLC) treatment (Table 2). Later, the tumor-cell inhibitory marker PD-L1 (CD274, previously known as B7-H1) was discovered in 1999-2000 [80] and PD-L2 (CD273, previously known as B7-DC) in 2001 [81] and was considered even much better control over immune cell checkpoint-based therapeutic targets [82] (Figure 2).

### 1.1. Connotation of Immune Checkpoint Markers

The significance of these immune checkpoint markers (PD-1, PD-L1/L2) as a ‘remarkable discoveries’ was initially ratified after the experiments in mouse models, suggesting the requirement of these markers are equally vital in maintaining immune homeostasis by regulating a balance between ‘immune response’ and ‘immune tolerance’ via its acceleratory/co-stimulatory (CD28) as well as inhibitory (PD-1, CTLA4) receptors. For example, (i) the immune inhibitory function of PD-1 was demonstrated by characterizing autoimmune phenotype in PD1-deficient (PD-1^−/−^) mice, suggesting the loss of peripheral tolerance [106]. (ii) lupus like arthritis and glomerulus-nephritis in PD-1^−/−^ C57BL/6 mice [107]. (iii) fatal myocarditis in PD-1^−/−^ Balb/c and MRL mice [108,109]. (iv) Type-I diabetes in PD-1^−/−^ NOD-mice [110,111]. (v) host vs graft disease in PD-1^−/−^ mice crossed with H-2LD-specific 2C-TCR transgenic mice [107], and (vi) hydronephrosis associated abnormalities in PD-1^−/−^ Balb/c mice [112]. Similarly, the first report on CTLA-4 blockade (negative regulator of T-cell activation [113]) in anti-tumor immunity was demonstrated in 1996 [114] and the first clinical report of CTLA-4 against melanoma in 2003 [115,116]. These discoveries were sufficient enough to encourage scientists to investigate its human relevance and further clinical trial (CT) studies. 

### 1.2. ICB Drug-Resistance and Toxicities 

Beside patient‘s age, cancer stage (I–IV) and various environmental factors; there might be several other factors for increased drug-resistance and reduced efficacy of ICB therapeutics [75]. For example, sub-optimal antibody dose, insufficient immune cell activation, intra-tumoral microenvironment, reduced memory cell formation and impaired effector cell functions after first course of treatment schedule. Sometimes, high-dose antibody toxicity also becomes a major concern for its adverse consequences (Figure 1). Therefore, a more advanced and unique therapy is required promptly to overcome this major issues. 

Conclusively, our study devotes to improve the efficacy of ICB-therapy by co-targeting (i) epitranscriptomics (ii) intracellular immune checkpoints and (iii) microRNAs. More importantly, our investigation would help to design a specialized approach or custom-made strategies to improve the efficacy of ICB-therapy [7,117,118]. 

## 2. Milestones in ICB therapeutics

### 2.1. Discovery of ICB Therapy

The invention of ICB-therapy was started after the discovery of PD-1, PD-L1 and CTLA-4 like immune checkpoint markers. Write after that, several other immune-based markers were tested against broad range of tumor types, covered extensively in these articles [119,120,121,122]. However, this section diagrammatically simplifies the milestone in the development of ICB-therapy and recent strategies to improve the efficacy of ICB-antibodies by combining personalized medicines (Figure 1).

### 2.2. Mechanism of ICB/ICI-Therapeutics

The detail mechanism of ICB-therapy including current drug-resistance issues was already well described by Wei and Allison et al., 2018 [123], Jenkins et al., 2018 [73], Kalbasi et al., 2020 [75] and Barrueto et al., 2020 [124]. However, this section briefly simplifies the understanding of immune checkpoint markers and its implications in developing therapeutic antibodies. Although, our main focus is to resolve the issues associated with ICB drug-resistances by promoting personalized therapy (Figure 2).

### 2.3. Strategies to Overcome ICB Drug-Resistance 

This section describes the strategies to overcome the issues mainly associated with drug-resistance and high-dose antibody toxicities. For example, (i) Epitranscriptomic approach: by targeting N^6^A modifiers: editor/writer, eraser/remover and effector/reader [125,126]. (ii) Bi-specific antibody approach: by co-targeting PD-1 and CD47 markers enlightened by ImmuneOncia therapeutics Inc. Korea [127,128] and AstraZeneca [129]. (iii) Antibody combination: by combining two antibodies targeting PD-1, PD-L1/L2 and CTLA-4 targets [123]. (iv) Precision medicines/personalized therapy: combining immunotherapy targeting intracellular immune checkpoints (CISH/SOCS-1) in specific immune cells [130,131]. (v) Molecular medicine: epigenetic modifiers targeting DNA, histone proteins and chromatin remodelers [22,132] and (vi) microRNAs [41,133,134] (Table 1 and Table 3, Figure 1). The detail of ICB-therapy and strategies to overcome ICB-drug resistance is well described in this review [75], however covering all is out of scope of this review.

## 3. Epitranscriptomics in ICB-Therapeutics

Epitranscriptomics has contributed greatly to the clinico-biological practices due to its diverse role in regulating at post-transcriptional and translational level. Epitranscriptomics generally referred to chemical modifications in the RNA molecule without changing the nucleotide sequence. So far more than 160 chemical modifications have been identified [151] playing a crucial role in regulating various biological processes, for example, in acute myeloid leukemia treatment [125], lung adenocarcinoma [152] gastric cancer [153] and broad range tumor types [151,154,155]. The major epitranscriptomic machineries (writer/editor, eraser/remover and readers/effector [156] not only regulate RNAs by specific regulatory mechanism [157,158] but also decide the fate of the cells and its associated immune disorders in cellular context-dependent manner. In this section, we have described the clinical application of epitranscriptomics in overcoming the issues associated with ICB drug-resistance by combining personalized approach.

### 3.1. Editors (Writers):

#### 3.1.1. Mettl-3/14 in Anti-PD-1 Resistance (Colorectal Cancer)

**Figure 3:**
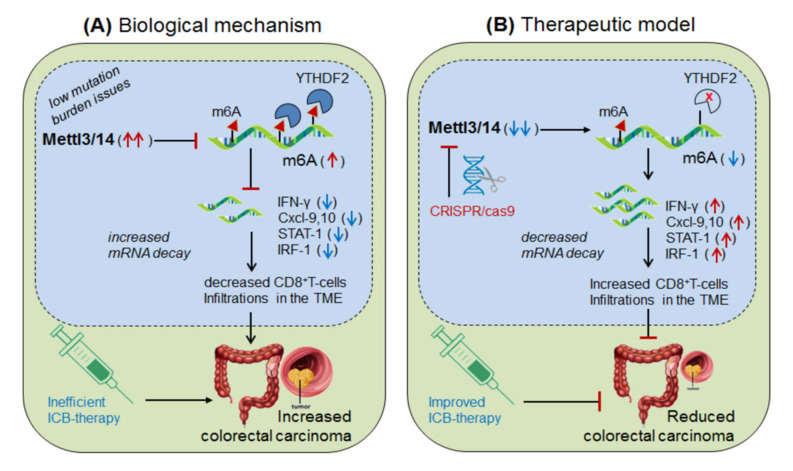
Therapeutic model targeting ‘Mettl-3/14’ in colorectal cancer. (**A**) Biological mechanism: Mettl-3/14 is up-regulated in colorectal cancer and melanoma, and inhibits the expression of IFNγ-STAT1-IRF1 signaling via YTHDF2-mediated (decreased mRNA decay) mechanism and thereby decreases the efficacy of anti-PD-1 effect by lowering CD8^+^T-cell infiltrations in the TME, and thus facilitated disease progression. (**B**) Therapeutic model: Anti-Mettl-3/14 therapy: CRISPR/cas9-silencing of Mettl-3/14 increases the expression of its target IFNγ-STAT1-IRF1 genes/signaling by reducing the recruitment of YTHDF2-mediated decay mechanism, and thus enhances the efficacy of anti-PD-1 antibody by increasing infiltrations of CD8^+^T-cell in the TME. Moreover, FTO overexpression might decrease Mettl-3/14 level via balancing mechanisms, and ‘anti-YTHDF2 therapy’ by directly augmenting target gene expressions, via its mRNA stability mechanisms, might have therapeutic benefits.

Wang, et al., 2020 [8] demonstrated the role of Mettl-3/14 (m6A-writer enzyme) in improving the efficacy of anti-PD-1 therapy. They found that even after standard anti-PD-1 treatment, still some patients with colorectal cancer and melanoma develop resistance, because of insufficient immune response generated by the tumors with low mutation burden issues (mismatch-repair-proficient or microsatellite instability-low ‘pMMR-MSI-L’) constituting ~85% of the patients [159]. They found that, these patients have significantly increased level of Mettl-3/14, which has impaired the function of certain crucial genes under the tumor microenvironment (TME). Interestingly, CRISPR/cas9-mediated deletion of Mettl-3/14 in colorectal cancer cell line (CT26) and murine melanoma cell line (B16) has not only increased cytotoxic CD8^+^T-cell (CTL) infiltrations in the TME but also provided durable adoptive immune response. Mechanistically, they justified that, the loss of Mettl-3/14 augmented mRNA-stability of IFNγ, STAT-1 and IRF-1 by promoting IFNγ-STAT1-IRF1-signalling through YTHDF2 reader proteins [157,158], leading to prolong secretion of these cytokines in the TME, resulting in strong immune response. These investigations suggest the key role of Mettl-3/14 in inhibiting the efficacy of anti-PD-1 therapy by decreasing IFNγ, Cxcl-9 and Cxcl10-mediated immune response. Conclusively, this study endorsed the immunotherapeutic potential of m6A-writer in improving the efficacy of anti-PD-1 antibody by silencing Mettl-3/14 in the TME [8]. Moreover, overexpressing FTO (m6A-demethylase) or by targeting intracellular YTHDF2 (m6A-reader protein) in decreasing Mettl-3/14 methylation could be considered as an alternative strategy to improve anti-PD-1 therapeutics (Figure 3). 

#### 3.1.2. Mettl-3 in Anti-PD-1 Resistance (Lung Metastasis)

**Figure 4:**
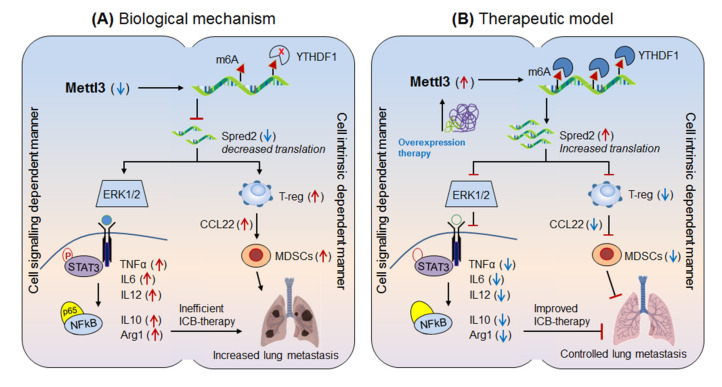
Therapeutic model targeting ‘Mettl-3’ in lung metastasis. (**A**) Biological mechanism: Mettl-3 is significantly down-regulated in tumor associated macrophage (TAM) and thereby alters M1/M2-macrophage-polarization and thus increases the infiltration of immunosuppressive populations (T-reg and MDSCs) in the tumor microenvironment, resulting in increased tumor growth and lung metastasis. (**B**) Therapeutic model: overexpression therapy: overexpression of Mettl-3 recruited YTHDF1-reader protein which increases the expression of its target ‘spred2’ gene, resulting in decreased infiltration of immunosuppressive cells by reducing ERK1/2 signaling, that finely reduces lung metastasis by improving the efficacy of anti-PD-1 therapeutics.

Yin et al., 2021 [9] demonstrated the molecular mechanism of anti-PD-1 resistance by Mettl3-mediated macrophage polarization, and enlightened the significance of decreased Mettl3-level in lung metastasis. *Yin and collogues* identified that the in vitro co-culture of bone marrow derived macrophages (BMDM) with B16 (skin melanoma) or LLC (lewis lung carcinoma) cell lines decreases the expression of Mettl-3. Moreover, the in vivo implantation of B16 and LLC cell lines into syngeneic mice also decreases Mettl-3 expression in tumor associated macrophages (TAM: CD11b^+^F4/80^+^), suggesting the loss of Mettl-3 in promoting tumor growth and thus survival defect. To investigate the underlying mechanism, they used specific mouse model selectively depleted with Mettl-3 by crossing Mettl3^f1/f1^ and Lyz2-cre mice. Interestingly, B16/LLC injected mice showed rapid tumor progression as well as lung metastasis in Mettl3-deficient (Mettl3^fl/fl^Lyz2^cre/+^ or Mettl3^cKO^) mice as compared to the wild type (Mettl3^fl/fl^Lyz2^+/+^ or Mettl3^WT^) mice. In addition, abnormal macrophage polarization characterized by increased M1-pro-inflammatory/anti-tumor (CD11b^+^F4/80^+^NOS_2_^high^IL-12^high^) and decreased M2-anti-inflammatory/pro-tumor (CD11b^+^F4/80^+^ARG1^high^IL-10^high^) were also noted in Mettl3^cKO^ mice, along with impaired response to effector T-cell functions. More importantly, the flow cytometry analysis of tumor bearing mice (TBM) revealed increased infiltration of immunosuppressive cells like, regulatory T-cells (T-reg: CD4^+^CD25^+^Foxp3^+^) and myeloid derived suppressor cells (MDSCs: CD11b^+^Gr1^+^) in the TME evidenced by increased expression of CCL22-migratory marker in Mettl3^cKO^ mice. Reciprocally, selective depletion of T-reg (anti-CD25) and macrophages (clodronate liposomes) significantly decreased both tumor growth and lung metastasis. This result clearly suggests that anti-PD-1 resistance has occurred due to (i) increased abundance of immunosuppressive populations. (ii) impaired CD8^+^ T-cell effector function and (iii) hyper-polarization of M1/M2-macrophage in the TME of Mettl3^cKO^ mice mimicking diseased model. Mechanistically, m6A-methylated RNA-immunoprecipitation followed by high throughput sequencing (MeRIP-Seq) of the RNA isolated from BMDMs from Mettl3^WT^ and Mettl3^cKO^ mice revealed ‘spred2’ as a potential downregulated target of Mettl-3 overlapping MAPK/ERK pathways. This suggests that spred2 is an upstream target of NFκB and STAT3 pathway in polarizing M1/M2-macrophage, as well as negative regulator of ERK/MAPK-signaling [160]. The above findings were further validated by reverting the M1/M2-polarizations by selective inhibition of STAT3 (S3I-201) and NFκB (BAY-11-7082) pathways, justified by chromatin immunoprecipitation (ChIP) for increased STAT3 binding to Arg1 promoter (M2-polarization marker) in Mettl3^cKO^. Next, with regard to epigenetic regulation, the overexpression of Mettl-3 increases the translation of ‘spred2’ by YTHDF1-mediated mechanism, confirmed by increased binding of YTHDF1 to spred2 via RNA-IP. Conversely, knockdown of YTHDF1 (siRNA) diminishes spred2 level. This result further supports ‘spred2’ as a target of Mettl-3 and is regulated by YTHDF1-mediated mechanism [126] rather than by targeting mRNA-stability or promoter-dependent translation mechanisms [161], and thereby activated ERK-mediated (being spred2 as a negative regulator of ERK signaling) other downstream signaling pathways in polarizing M1/M2-macrophages. Additionally, polysome profiling for translation-active (>80S) regulatory site and m6A-conserved motif ‘GGAC’ analysis further authenticate spred2 regulation by Mettl3-methylation mechanisms, validated by decreased spred2 expression in mutant (GCTC) as compared to the wild-type (GGAC) motif. Lastly, the link between Mettl3-driven spred2 and ERK1/2-NFκB-STAT3 signaling confirms the polarization of M1/M2-macrophage by aggravating TNFα and IL-6 (M1: pro-inflammatory) and IL-10, Arg1 (M2: anti-inflammatory) cytokines, validated by diminished expression of the same by selective signaling inhibitors. Taken together, these results suggest the crucial role of Mettl-3 in impairing anti-PD-1 efficacy by (i) polarizing M1/M2-macrophage via activating spred2-mediated ERK1/2-NFκB-STAT3 signaling cascade through cytokine milieu and (ii) by recruiting immunosuppressive cell populations in the TME. Conclusively, Mettl-3 is key player in reducing the efficacy of anti-PD-1 therapy, and therefore targeting (overexpressing) Mettl-3 could be a promising approach to control cancer metastasis by enhancing the efficacy of anti-PD-1 antibodies (Figure 4) [9]. This hypothesis was further supported by Yi, et al., 2020 in regulating PD-L1 mediated HNSCC control by implicating m6A-modifiers, and thus potentiating its therapeutic value by targeting G2M checkpoint, mTORC1 and PI3K/AKT/mTOR signaling analyzed via cancer genome atlas TCGA (n = 499) and GSE65858 (n = 270) cohorts [10].

### 3.2. Erasers (Removers):

#### 3.2.1. FTO in Anti-PD1 Resistance (Melanoma): 

**Figure 5:**
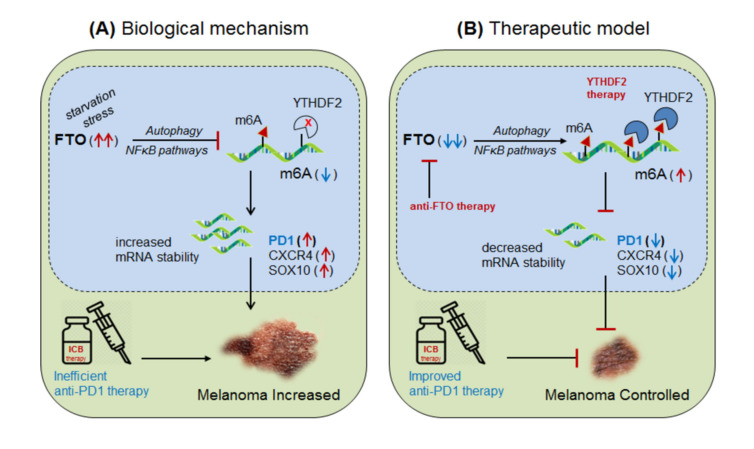
Therapeutic model targeting ‘FTO’ in melanoma. (**A**) Biological mechanism: FTO is highly up-regulated in melanoma (due to starvation stress through NFκB-pathways and autophagy) leading to increased mRNA transcript of the critical pro-tumorigenic genes (PD-1, CXCR4 and SOX10) by decreasing m6A-methylation mark, resulting in increased melanoma progression. (**B**) Therapeutic model: (i) Anti-FTO therapy: selective inhibition of FTO (FTO inhibitor [125]) or intracellular silencing of ‘FTO’ controls melanoma progression by selectively increasing the methylation-inhibition of its pro-tumorigenic genes, including PD-1 immune checkpoint markers by increasing the efficacy of anti-PD-1 antibody [163]. (ii) YTHDF2 therapy: YTHDF2 overexpression would control melanoma progression by accelerating the mRNA-decay of critical pro-tumorigenic genes. Moreover, targeted overexpression of Mettl-3 might control melanoma progression by destabilizing critical tumor-promoting genes by recruiting YTHDF2-reader proteins. Furthermore, targeting NFκB/mTOR signaling might also control melanoma progression by limiting nutrient supply to the tumors.

Yang et al., 2019 [11] demonstrated the role of m6A-eraser protein ‘FTO’ in melanoma progression, a type of skin cancer, and enlightened the intrinsic mechanism to improve the efficacy of anti-PD-1 therapy by targeting FTO. *Yang and colleagues* found that FTO is significantly up-regulated in human melanoma patients (metastatic skin samples n = 65) including human (Mel624) and mouse (B16F10) cell lines, and facilitated rapid tumorigenesis, caused by metabolic starvation stress in mice requiring autophagy and NFκB pathway [162]. However, selective depletion of ‘FTO’ not only increases sensitivity to anti-PD-1 therapy but also increases m6A methylation-inhibition of critical pro-tumorigenic (tumor-promoting) genes. Mechanistically, they proved that FTO-deficiency increases m6A-methylation at 5′UTR and 3′UTR of target genes; PD-1 (PDCD1), CXCR4 and SOX10, and thereby causing rapid mRNA-degradation by recruiting YTHDF2-reader proteins [155,157,158], confirmed by YTHDF2-knockdown in ‘increasing’ and YTHDF2-overexpression in ‘decreasing’ melanoma growth. Moreover, FTO-deficiency enhances the sensitivity of anti-PD-1 treatment by IFNγ-mediated cytokine response. These results clearly suggest that FTO plays a crucial role in melanoma tumorigenesis by regulating mTOR signalling through limiting the nutrient supply to the tumours [162]. Therefore, co-targeting FTO in combination with ICB-antibodies would be a promising approach to control melanoma progression [11]. This hypothesis was also supported by Singh et al., 2016 in controlling triple-negative inflammatory breast cancer cells using FTO (MO-I-500) inhibitor [125,163,164]. Theoretically, targeted overexpression of Mettl-3 might also control melanoma progression by decreasing FTO via balancing mechanism, and also by directly inhibiting the expression of pro-tumorigenic genes via recruiting YTHDF2 reader proteins (Figure 5).

#### 3.2.2. FTO in Anti-PD-1 Resistance (Colon Cancer)

Tsuruta et al., 2020 [12] demonstrated the role of FTO in colon cancer progression and enlightened the molecular mechanism to control cancer carcinogenesis by targeting FTO. They found that FTO is aberrantly expressed in colon cancer cell line (HCT-116). Moreover, immune checkpoint molecule ‘PD-L1’ expression was also highly up-regulated. Therefore, targeting FTO by selective depletion (siRNA) not only reduced FTO-level but also significantly decreased PD-L1 expression in IFNγ signaling-independent manner at both mRNA and protein levels. This result clearly suggests that FTO facilitates colon cancer progression by promoting the expression of PD-L1 markers. Mechanistically, they proved (via RNA immunoprecipitation) that FTO binds to m6A-marked PD-L1 mRNA and elevates its expression probably by decreasing mRNA-decay mechanism. Taken together, this study reveals the critical role of FTO in facilitating colon carcinoma by increasing PD-L1 expression, and therefore targeting FTO by means of either selective FTO inhibitor [125] or CRISPR/Cas9-based methods could hold the potential to control colon cancer in combination with anti-PD-L1 therapeutics (Table 1) [12].

#### 3.2.3. ALKBH5 in Anti-PD-1 Resistance (Melanoma) 

**Figure 6:**
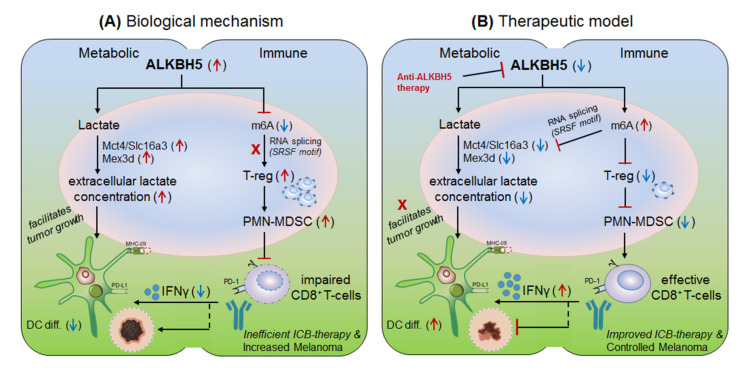
Therapeutic model targeting intracellular checkpoint ‘ALKBH5’ in melanoma. (**A**) Biological mechanism: The ALKBH5 abnormally expressed in melanoma and colorectal cell carcinomas, and impairs the efficacy of anti-PD-1 therapy by (i) recruiting immunosuppressive; regulatory T-cell (T-reg) and polymorphonuclear myeloid derived suppressor cells (PMN-MDSC) abundances in the TME. (ii) by impairing DC-differentiation resulting in decreased CD8^+^T-cell effector functions. (iii) by increasing extracellular lactate availability to the tumors by up-regulating the expression of Mct4/Slc16a3 genes due to decreased m6A-methylation mark associated mechanism. (**B**) Therapeutic model: (i) Anti-ALKBH5 therapy: selective inhibition of ALKBH5 [168] by increasing m6A methylation-mediated inhibition of crucial genes essential to increase the efficacy of CD8^+^T-effector cells. (ii) T-reg/PMN-MDSCs depletion therapy: could also show the therapeutic propensity by rescuing the immunosuppressive environment. (iii) Increasing DC-differentiation: could be also a promising approach to enhance DC-mediated CD8^+^T-cell effector function. (iv) Targeting metabolic genes: could be an alternative approach to control melanoma tumorigenesis by limiting extracellular lactate accumulation in the TME. Collectively, all these approach seems promising in overcoming the issues associated with ICB drug-resistance.

Li et al., 2020 [13] explained the role of another m6A-eraser protein ‘ALKBH5’ in progression of melanoma-associated metastatic cancer, and enlightened the molecular mechanism to overcome anti-PD-1 resistance by targeting ALKBH5. Based on their previous studies [8] for the role of Mettl-3/14 in melanoma progression, the authors hypothesized that ALKBH5 might also have significant role in regulating the efficacy of anti-PD-1 therapeutics. To this end, *Li and colleagues* used B16 (mouse melanoma) and CT26 (colorectal carcinoma)-induced TBM model, and selectively depleted ALKBH5 and/or FTO (CRISPR/Cas9-mediated silencing) in B16 and CT26 cell lines respectively, and injected subcutaneously into wild-type C57BL/6 and BALB/c mice to create tumor, followed by 1-day prior vaccination with irradiated B16 cells secreting GM-CSF ‘GVAX’ to induce sufficient antitumor T-cell response, and finally anti-PD-1 antibody treatment was given to check its efficacy. Interestingly, ALKBH5^−/−^ TBM showed prolonged survival and slower tumor growth as compared to the non-transfected (NTC) control mice, suggesting the direct involvement of ‘ALKBH5’ in interfering with the efficacy of anti-PD-1 antibody. To further elucidate the role of ALKBH5 in modulating GVAX/anti-PD-1 treatment, they analysed tumor infiltrating lymphocytes (TILs) by FACS and found that among total CD45^+^CD4^+^CD8^+^ gated populations, ALKBH5^−/−^ mice have elevated granzyme-B (GZMB)^+^CD8^+^, GZMB^+^CD4^+^ T-cell, NK-cell (CD56^+^) and dendritic cell (DCs: CD45^+^Ly6C^-^MHC-II^+^CD24^hi^F4/80^lo^) numbers, but more importantly, T-reg (CD4^+^Foxp3^+^) and polymorphonuclear myeloid derived suppressor cell (PMN-MDSCs: CD45^+^CD11b^+^Ly6G^+^Ly6C^lo^F4/80^−^MHC-II^−^) populations were drastically reduced as compared to the control mice. This was further validated by immunohistochemistry staining (IHC) of the MDSC-mLy6G, however, no differences in other immune cell populations (MDSC and macrophage) were noted. This suggests that ALKBH5 has the potential to recruit immunosuppressive (T-reg and PMN-MDSCs) populations in the TME during ICB therapy. Again, to stamp the selective function of immunosuppressive cells in inhibiting anti-PD-1 effect, they specifically depleted T-regs (anti-CD25) and PMN-MDSCs cells in the NTC control mice, resulting in delayed tumor progression as compared to the ALKBH5^−/−^ model (due to already fewer T-reg numbers), confirming the immunosuppressive function of T-regs (induced by MDSCs) in impairing the efficacy of anti-PD-1 antibody by inhibiting CD8^+^T-cells effector functions through decreasing DC-differentiation (CD45^+^Ly6C^-^MHC-II^+^CD24_hi_F4/80^lo^) markers [165]. These observations clearly suggest that ALKBH5 recruits immunosuppressive populations in the TME and thereby interfering with the efficacy of anti-PD-1 therapy. Next, to identify the molecular targets, they sequenced RNA isolated from ALKBH5/FTO^−/−^ B16 tumors and compared it with the NTC-control TBM on day-12 after GVAX/anti-PD1 treatment. Interestingly, the gene ontology (GO) analysis of the differentially expressed genes (DEG) revealed ALKBH5 is associated with metabolic genes especially ‘Mct4/Slc16a3’ involved in lactate metabolism, whereas, FTO is associated with IFNγ and chemokine signalling pathways. This was validated by increased IFNγ intermediates (qRT-PCR expression) upon in-vitro stimulation of IFNγ to the FTO^−/−^ B16 cells. Moreover, the comparison of mouse DEGs with human melanoma patients (n = 21 anti-PD1 therapy responder) and (n = 17 non-responder) reveals eight common genes associated with ALKBH5-deficiency and eleven common genes with FTO-deficiency, indicating ‘conserved’ and potential targets of ALKBH5 and FTO in mouse as well as human receiving anti-PD1 therapy. This suggests that ALKBH5 modulates anti-PD-1 resistance by recruiting immunosuppressive T-reg cells and by modulating metabolic genes whereas FTO works by targeting IFNγ and by modulating inflammatory chemokine-mediated signalling pathways in the TME. Next, epigenetic analysis via LC-MS/MS reveals higher m6A-abundance in ALKBH5-deficient as compared to FTO-deficient B16 tumours, which meaningfully suppresses the expression of m6A-mediated ‘Mct4/Slc16a3’ in ALKBH5 alone and ‘Mex3d’ in ALKBH5 and FTO both. Moreover, MeRIP-seq reveals enriched SRSF motif (a subunit of SAG core involved in RNA splicing [166]) in ALKBH5-deficient tumors as compared to FTO, suggesting different mechanisms of action of these two de-methylases in modulating anti-PD1 efficacies. Collectively, these results suggest that ALKBH5 and FTO target metabolic genes and increase the expression of Mct4/Slc16a3 and Mex3d (supplementing lactate to the tumour) by inhibiting m6A methylation-mediated RNA-splicing mechanisms, supported by Zaho et al., 2014 [167], (Figure 6, therapeutic model). Furthermore, to dig out the m6A-modulated genes via RNA-splicing mechanism, they identified m6A-enriched transcripts around 5′-3′ splice sites by m6-CLIP and found the involvement of three immunotherapeutic resistance genes Eif4a2, Arid4b and USP15 affecting the response of anti-PD-1 therapeutics by regulating transcription, translation and T-reg activation via TGF-β signalling in the TME. (Figure 6) Taken together, ALKBH5 is playing a crucial role in promoting tumour metastasis, and therefore intracellular silencing of ALKBH5 in the TME would hold the potential to control tumor metastasis via increasing the efficacy to anti-PD-1 therapeutics [13].

### 3.3. Effectors (Readers):

#### 3.3.1. YTHDF1 in Anti-PD1 Resistance (Solid Tumors)

**Figure 7:**
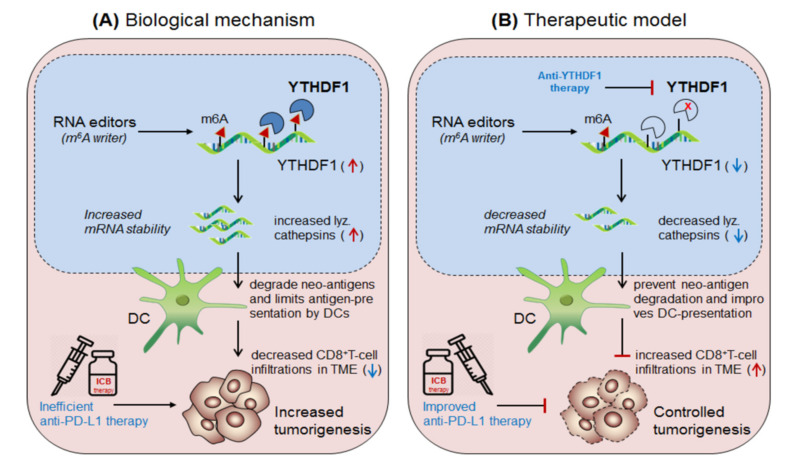
Therapeutic model targeting intracellular checkpoint ‘YTHDF1’ in enhancing DC-mediated anti-tumor immunity. (**A**) Biological mechanism: The ‘YTHDF1’ reader protein recognizes m6A-marked cathepsin transcript and increases it’s mRNA and protein level, which translocate into the phagosome and degrades neo-antigens, and thus limiting its recognition and cross-presentation by the DCs, and thereby the impaired DCs decreases CD8^+^T-cell effector function, leading to decreased efficacy of anti-PD-1 therapy, resulting in increased tumor growth. (**B**) Therapeutic model: Anti-YTHDF1 therapy: inhibits cathepsin level and thus unable to degrade neo-antigens, resulting in effective antigen recognition and cross-presentation by DCs and thereby enhanced CD8^+^T-cell effector function which improves the efficacy of anti-PD-1 therapy.

Han et al., 2019 [14] demonstrated the synergistic role of dendritic cells expressing ‘m6A-writer’ and ‘YTHDF1-readers’ proteins in anti-tumor immunity. They found that despite the presence of numerous neo-antigens, some patients still failed to generate sufficient anti-tumor response. To this end, in discovering the intrinsic molecular mechanism, they generated dendritic cell-specific conditional knockout mice depleted with YTHDF1 (YTHDF1^cKO^) gene. Surprisingly, the loss of YTHDF1 enhances antigen-recognition and cross-presentation ability of DCs in-vivo, resulting in elevated CD8^+^ T-cell infiltration in the TME as compared to the control wild type (YTHDF1^WT^) mice. Moreover YTHDF1^cKO^ mice showed enhanced response to anti-PD1 therapy [159]. Mechanistically, they proved that the wild type mice, in the presence of m^6^A mRNA-methylation machineries recruited YTHDF1 reader proteins at the lysosomal-cathepsins mRNA axis, resulting in increased mRNA-stability, and thereby increased the abundance of cathepsin proteins in the phagosomal compartments of the DCs, causing severe degradation of the neo-antigens and thus limiting the antigen availability to the DCs for antigen-recognition and further cross-presentation to CD8^+^T-cells in the cytosol. This result suggests that YTHDF1 is playing a crucial role in suppressing anti-tumor immunity [14], and therefore intracellular silencing of YTHDF1 in DCs designates its potential to enhance anti-tumor immunity. Collectively, this discovery reveals two important mechanisms to enhance anti-tumor immunity by co-targeting (i) anti-YTHDF1 therapy: where, YTHDF1-deficiency protect ‘antigen-degradation’ and allows efficient recognition and presentation by DCs, in-turn, further increases the abundance of DC-mediated effector CD8^+^T-cells by cross-presentation mechanism, supported by Ding et al., 2021 [169] and (ii) by enhancing anti-PD-1/PD-L1 efficacy: which further potentiates the efficacy of anti-PD-1 immunotherapy by enhancing the effector function of CD8^+^T-cells in the TME [159] Figure 7.

#### 3.3.2. YTHDF2 in Anti-PD1 Resistance (Brain Tumors)

Lin et al., 2020 [15] demonstrated the role of YTHDF2 in progression of lower-grade glioma (LGG) also called ‘pilocytic astrocytoma’, a type of early stage brain tumor. They showed that YTHDF2 is abnormally expressed in various types of cancers and reduces overall longevity and survival. The higher expression of YTHDF2 has been positively correlated with immune cell (B-cells, T-cells, DCs, MΦ and neutrophils) expressing PD-1, TIM-3 and CTLA-4 markers. Therefore, targeting YTHDF2 in DCs would hold the potential to enhance anti-tumor immunity in combination with ICB-therapeutics [14,15]. A similar pre-clinical trial was proposed by jubilant-therapeutics targeting PD-1 inhibitor (with brain penetrant PRMT5) in controlling LGG, potentiating the scope to utilize in combinations with immune cells targeting intracellular checkpoints as targeted therapy. Moreover, Garzon-Muvdi et al., 2018, have supported the prominence of DC activation in enhancing the efficacy of anti-PD-1 immunotherapy against glioblastoma [16]. Taken together, this study reveals the importance of targeting YTHDF2 in combination with DC-immunotherapy [7] to enhance the efficacy of ICB therapy against early stage brain tumors [15], (Table 3).

## 4. Immune Cells: Targeting Intracellular Checkpoint ‘CISH’ in Combination with ICB-Therapeutics and Recent Clinical Trials

Cytokine-inducible SH2-domain containing protein (CISH or CIS) is one of the eighth members of SOCS family of proteins, recently gaining high attention due to its widespread regulatory role in cytokine signalling [170,171] and its involvement in more than 349-diseased (https://platform.opentargets.org/target/ENSG00000114737/associations; accessed on: 25 July 2021) [172,173,174] phenotypes. The therapeutic significance of ‘CISH’ can be evidenced by a recent clinical trial (NCT04426669, NCT03538613 by Intima Bioscience, UK; and ONKT102, ONKT103 and ONKT104 by ONK therapeutics, Ireland) targeting NK-cells, TILs and DCs for the treatment of broad range of metastatic cancers [1,2]. The so-called personalized medicine targeting ‘CISH’ in immune cells has shown promising effect in improving the efficacy of ICB-therapeutics [3,5]. Therefore, this section highlights another layer of strengthening ICB-therapy by targeting intracellular immune checkpoint ‘CISH’ in different immune cells. A few important links/references are also provided in (Box-2) supporting ‘CISH/SOCS’ to be used as potential markers in developing personalized medicine [7,175,176,177,178] (Table 3, Box-1). 

### 4.1. NK-Cells Targeting CISH in ICB Therapeutics

**Figure 8:**
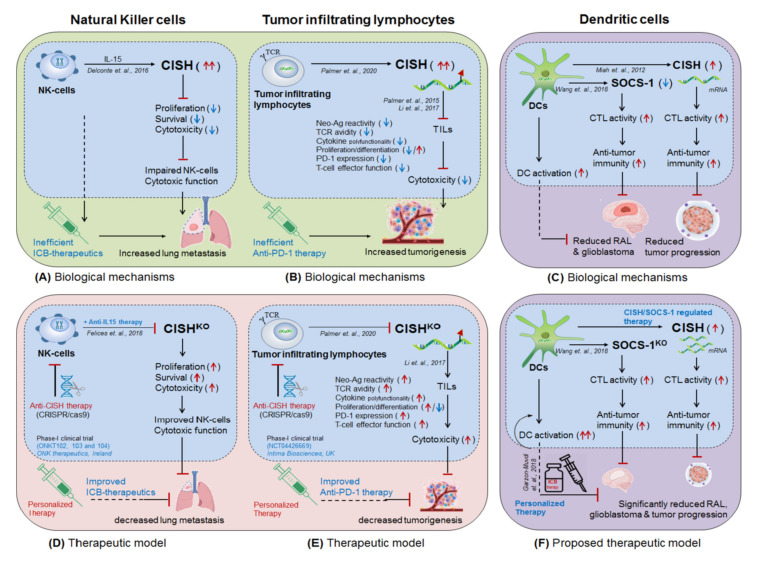
Immunotherapeutic model co-targeting intracellular checkpoint (CISH/SOCS-1) in combination with ICB-therapeutics. (**A**) NK-cell biological mechanism: CISH expression in natural killer cell impairs its cytolytic function by reducing NK-cell proliferation, fitness and survival inside the TME [190] and thus insufficient in protecting lung metastasis [3]. (**B**) Tumor infiltrating lymphocytes (TILs) biological mechanism: CISH expression in TILs also impairs T-cells function by reducing TCR avidity, cytokine poly-functionality and CD8^+^T-cells effector functions and thereby facilitating tumorigenesis [6]. (**C**) Dendritic cell biological mechanism: SOCS1-deficiency and CISH-expression in DCs has been proven in regulating DC-mediated anti-tumor immunity against broad range of solid tumors by increasing DC-activation and CTL-activity [7,184]. (**D**) Therapeutic model targeting CISH in NK-cells: (i) anti-CISH therapy: CISH deletion (CRISPR/Cas9) in NK-cells improves overall NK-cell survival, proliferation, fitness and effector functions, and thereby potentiating its cytotoxic activity. (ii) Personalized therapy: co-targeting CISH^−/−^ NK-cells with ICB-antibodies significantly improves ICB-efficacy in controlling lung metastasis [190]. A relevant human clinical trial (ONKT102, ONKT103 and ONKT104) targeting CISH-deficient NK-cells has been proposed by ONK therapeutics, Ireland, against hematological malignancies and solid tumors [135] (Box-1). (**E**) Therapeutic model targeting CISH in TILs: (i) anti-CISH therapy: CISH deletion (CRISPR/Cas9) in TILs improves T-cell effector functions by increasing TCR avidity and cytokine poly-functionality, and thereby potentiating its effector function. (ii) Personalized therapy: co-targeting CISH^−/−^ TILs with ICB-antibodies significantly improves ICB-efficacy in increasing anti-tumor immunity. A relevant human clinical trial (NCT04426669, NCT03538613) targeting CISH^−/−^ TILs has been proposed by Intima Bioscience, Inc. UK, against solid tumors and metastatic gastrointestinal cancers [1,2] (Box-1). (**F**) Proposed therapeutic model targeting SOCS-1/CISH in DCs: (i) SOCS-1/CISH regulated therapy: Regulation of SOCS-1/CISH in DC-mediated anti-tumor immunity has been already shown in increasing CTL-activity against broad range of solid tumors [7,182,183,184], however, (ii) Personalized therapy: Co-targeting SOCS-1/CISH in DCs in combination with ICB-antibodies would further potentiates the efficacy of ICB-therapy, and therefore would be efficient in solving the issues associated with high-dose antibody toxicity and drug-resistance. A relevant human clinical trial (NCT01956630) targeting SOCS1^−/−^ in DCs has been conducted by academy of military medical sciences, China against leukemia [7] (Box-1) Moreover, a future research targeting epitranscriptomic machineries (m6A-modifiers) and microRNAs might further potentiate DCs by regulating CISH diversity [191], (Table 1).

Delconte et al., 2016 [3] demonstrated the therapeutic benefit of anti-PD-1 and anti-CTLA-4 antibodies in combination with intracellular checkpoint targeting CISH (CISH-deletion) in NK-cells for the treatment of lung metastasis and melanomas in murine model. They showed that intravenous (i.v.) administration of melanoma cell line (B16/F10) and prostate cancer cell line (RM-1) into CISH-deficient (CISH^−/−^) NK-cells have significantly reduced melanoma growth and metastatic nodule formation as compared to the wild-type (CISH^+/+^) mice, indicating the critical role of CISH in NK-cell cytotoxicity. The specificity of NK-cell function was confirmed by selective depletion of NK-cells (anti-asiolo GM1) in rendering susceptibility to B16F10 metastasis in CISH^−/−^ mice. Moreover, the adoptive transfer of CISH^−/−^ NK-cells into NK-cell^−/−^ recipient mice (Mcl1^f/f^ Ncr1-i^Cre^) showed fewer B16F10 metastases as compared to the mice receiving CISH^+/+^ NK cells. These results clearly suggest that (i) CISH is playing a crucial role in NK-cell activation. (ii) CISH is a negative regulator of NK-cell cytotoxicity and (iii) CISH^−/−^ NK cells are intrinsically more active. Moreover, combining anti-PD-1 and anti-CTLA-4 antibody treatment with CISH^−/−^ NK-cells drastically reduced lung metastasis as compared to the IgG control and CISH^+/+^ NK-cells alone in the adoptive transfer model, highlighting the potential therapeutic benefit that could be achieved when anti-PD-1 and anti-CTLA-4 therapy was combined with loss of CISH function. A similar result targeting intracellular checkpoint ‘CISH in NK-cells’ in combination with ICB-antibodies in increasing anti-tumor immunity was described by Putz et al., 2017 [4], Bernard et al., 2021 [179], Felices et al., 2018 [180] and Andre et al., 2018 [181]. Furthermore, a recent phase-I clinical trial (ONKT102, ONKT103 and ONKT104) targeting CISH-deletion in NK-cells was proposed by ONK therapeutics, Ireland, until 2021𠄲2022 against hematological malignancies (multiple myeloma and acute myeloid leukemia) and solid tumors (ovarian, NSCLC and breast cancers) [127,135,142] (Figure 8A,D). In addition to ‘CISH’, other immune checkpoint markers in NK-cells were nicely described by Chiossone et al., 2018 [121].

### 4.2. T-cells Targeting CISH in ICB Therapeutics

Palmer et al., 2020 [5] demonstrated the improved efficacy ICB-antibodies when combined with CISH-depleted (CISH^−/−^) TILs. *Palmer and colleagues* showed that the adoptive transfer of neoantigen specific TILs, derived from antigen expressing tumors, was failed in constantly eliciting durable tumor regression. Moreover, an altered expression of CD39, Tox and PD-1 marker was observed, suggesting the impaired function of effector CD8^+^T-cells in the TME. Interestingly, depletion of CISH (CRISPR/Cas9) in TILs significantly improved neoantigen recognition, TCR avidity, T-cell activation/expansion and tumor cytolysis, resulting in rapid-control over tumorigenesis. However an increased expression of PD-1 marker was also observed. Thus, co-targeting CISH^−/−^ TILs in combination with anti-PD1 antibody has proficiently controlled the tumor progression. This result clearly suggests the negative regulatory role of CISH in impairing T-cell effector functions, supported previously by Palmer et al., 2015 [6] and Periasamy et al., 2011 [147]. Therefore, co-targeting CISH^−/−^ TILs in combination with ICB-therapy would hold the potential to control tumor progression by improving the efficacy of ICB-antibodies as well as CD8^+^T-cell effector function. A relevant human Phase-I/II clinical trial (NCT04426669, NCT03538613) targeting CISH^−/−^ TILs in combination with ICB-therapy was proposed by Intima Bioscience, UK, until 2021𠄲2022 against wide range of tumor types and gastrointestinal cancer [1,2,115] (Figure 8B,E, Table 3, Box-1). 

### 4.3. Dendritic Cells Targeting SOCS-1/CISH in ICB-Therapeutics

Wang et al., 2018 [7] demonstrated the therapeutic benefit of targeting intracellular checkpoint SOCS-1, *one of the members of CISH family*, in DCs (SOCS-1^−/−^ DCs [182]) in controlling relapsed acute leukemia (RAL). They showed that the adoptive transfer of genetically modified DCs plus CIK cells is safe & effective in prolonging the survival of RAL patients (n = 48), by increasing DC activation, DC-maturation and TAA-induced CTL response. A relevant human phase-I/II clinical trial (NCT01956630) was conducted by the academy of military medical sciences, China and recommends it safe in-use [7]. A similar result was observed by Shen et al., 2004 in increasing anti-tumor immunity by silencing SOCS-1 in DCs [183]. More relevantly, Miah et al., 2012, demonstrated the importance of CISH-expressing DCs in increasing anti-tumor immunity by enhancing CTL activity in CISH^−/−^ CD11c mouse model [184], however it would be interesting to further investigate the role by combining ICB-antibodies. These findings suggest that targeting intracellular checkpoints ‘CISH/SOCS-1 in DCs’ would hold the potential to treat several cancers even-in-combination with ICB-therapeutics [7,185] (Figure 8C,F and Table 1 and Table 3).

In addition, several researches support the improved efficacy of DC-immunotherapy when combined with ICB-antibodies. For example; Zhang et al., 2019 [186] demonstrated the role of PD-1 blockade in increasing anti-tumor activity of specific DCs called DC-stimulated cytokine-induced killer cells (DC-CIK) generated in presence of anti-CD3 antibody, IFNγ, poly-hydroxyalkanoates and IL-2; characterized by co-expression of CD56 and CD3 or CD3 and CD8 markers. The authors have shown that the adoptive transfer of pre-treated DC-CIK with PD-1 inhibitor (Pembrolizumab) block PD-1/PD-L1 axis and therefore increased its cytotoxic activity as compared to the null-DCs. Moreover, an increased infiltration of effector CD8^+^T-cells was noted in a nude mouse xenograft model with hepatocellular carcinoma (HCC), resulting in reduced tumor growth. This study suggests the improved efficacy of pre-treated ‘PD-1 inhibitor DC-CIK’ in controlling HCC recurrence [186]. Similarly, Lim et al., 2016 supported the above hypothesis and further emphasized the anti-tumor activity of PD-1^−/−^ DCs in controlling HCC [187]. They showed that PD-1 expression on DCs reduces T-cell proliferation and suppresses CD8+T-cells effector function, resulting in decreased anti-tumor immunity. The adoptive transfer of PD-1^−/−^ DCs increases CD8^+^T-cells infiltrations along with IFNγ, IL-2, perforin and GZMB secretions in the TME and thereby causing rapid tumor control. This result suggests the improved efficacy of PD-1^−/−^ DCs in controlling HCC [187]. Next, Garzon-Muvdi et al., 2018 showed improved efficacy of anti-PD-1 when given in combination with DC-immunotherapy in controlling glioblastoma [16]. They showed that DC-activation through TLR3 agonist increases anti-tumor immunity in vitro. Moreover, TLR3 agonist poly (I:C)-injected mice showed increased DC-activation, antigen presentation and T-cell proliferation and thus enhancing the efficacy of ICB- therapy against glioblastoma [16]. 

Furthermore, Peng et al., 2020 [188] demonstrated the role of PD-L1 (ligand for PD-1) in the impairment of DCs. They showed that in response to antigenic exposure and IFN-II, type-I DCs (cDC1) increases the expression of PD-L1 and suppress CTL activity. Interestingly, the blocking of PD-L1 significantly improves DC-mediated T-cell infiltration and killing abilities in vitro. This result clearly suggests that PD-L1 expression is playing a crucial role in cDC1-impairment, and therefore targeting PD-L1 would hold the potential to enhance therapeutic benefits [188]. Similarly, Go et al., 2021 underlined the role of PD-L1-expressing DCs in reducing *helicobacter*-induced gastritis [189]. *Go and colleagues* showed that the treatment of anti-PD-L1 or PD-L1^−/−^ in bone marrow transplantation enhances gastritis. Upon a closer look, the loss of Ftl3 (Flt3^−/−^) or Zbtb46-diphtheria toxin receptor (DTR) mice showed decreased DC-abundances causing severe mucosal metaplasia, and suggesting the protective role of PD-L1 expressing DCs in controlling gastritis [189]. Furthermore, miRNA-200b and miRNA-152 have been found to be downregulated in HP-induced gastric cancer tissues, suggesting a negative correlation of miRNA-200b and miRNA-152 in B7-H1 (PD-1) expression. Therefore, targeting miRNA (miRNA-restoration or miRNA-mimics) would have therapeutic benefit against gastric cancer [57] (Table 1). Collectively, these investigations suggest the potential therapeutic benefit of targeting intracellular immune checkpoint ‘CISH/SOCS-1’ in enhancing the efficacy of ICB-therapy if combined with DC-immunotherapy (Table 3, Figure 8C,F).

## 5. MicroRNAs and Epigenetic Modifiers (DNA and Histone Proteins) in ICB-Therapy

### 5.1. MicroRNAs in ICB-Therapeutics

In addition to co-targeting epitranscriptomics, intracellular immune checkpoints (m6A-modifiers, CISH/SOCS-1) and microRNA can be another potential targets to overcome the issues associated with ICB-drug resistances and high-dose antibody toxicities. The significance of ‘microRNAs’ also cannot be ignored because of their versatile roles in regulating numerous genes associated with immune checkpoint inhibitors. *Kumar and colleagues* have extensively described the therapeutic potential of microRNAs in treating DC-mediated Th1/Th2-associated immune disorders [41,192]. However, this section highlights some of the recent advancements in the utilization of ‘microRNAs’ in improving the efficacy of ICB-therapeutics. For example, miR-21, miR-34, miR-146a, miR-155 including many others miRNAs (Table 3) [193,194] have shown astonishing results by targeting PD-1, PD-L1 and CTLA4 immune markers summarized well in these references [132,133]. 

### 5.2. Epigenetic Modifiers (DNA and Histone Proteins) in ICB-Therapeutics

Like microRNAs, other epigenetic modifiers such as DNA and histone modifiers also hold great potential to increase the efficacy of ICB-therapeutics. Therefore, this section summarizes in brief about the systemic utilization of DNA-modifiers alone or in combination with other immunotherapeutic procedures. The DNA modification machineries, also known as writers/editors: DNMTs; removers/erasers: TET-proteins; readers/effectors: histone proteins HATs (acetylases) & HDACs (de-acetylases) [195] and chromatin remodelers: SWI/SNF chromatin remodelling complexes [196,197] have significant role in modulating the genes associated with immune checkpoint markers. Collectively, section-5 nurtures the potential of DNA-epigenetic modifiers and microRNAs in developing efficient molecular medicines in resolving the issues associated with ICB-drug resistance and toxicities [198,199,200,201] (Table 3).

## 6. Biopharmaceutical Companies Developing Personalized Medicines: Targeting Intracellular Checkpoint ‘CISH’ in Combination with ICB-Therapeutics and Recent Clinical Trials

In this section, we have described some of the biopharmaceutical/cell-therapy companies entering into developing personalized medicines by targeting immune cells expressing ‘intracellular checkpoint CISH’ in combination with ICB-antibodies to overcome the issues associated with ICB drug-resistance and high-dose antibody toxicities in several caners. 

### 6.1. ONK Therapeutics Limited

ONK therapeutics is an Ireland-based cell therapy company, founded in 2015, conducting phase-I clinical trial against multiple myeloma, NSCLC and AML by targeting intracellular checkpoint ‘CISH’ in NK-cells. It was disclosed that the deletion of CISH improves the cytotoxic activity of NK-cells and therefore can be used efficiently to enhance the efficacy in combination with ICB-therapeutics. The respective phase-I clinical trials (ONKT102, ONKT103 and ONKT104) are estimated to complete until 2021𠄲2022 (https://www.onktherapeutics.com/pipeline; accessed on: 25 July 2021). A relevant patent (US10034925B2 and EP3434762A1) was also filed for securing global license to use CISH knockout NK-cells from Australia’s WEHI” on 28 May 2021 (www.onktherapeutics.com; accessed on: 25 July 2021) [135]. 

### 6.2. Intima Bioscience, Inc.

Intima Bioscience is a UK-based Biotechnology Company, founded in 2021, conducting phase-I/II clinical trials (NCT04426669) against metastatic gastrointestinal (GIT) cancer patients by administering CISH-inactivated TILs by CRISPR/Cas9 system [1]. It is estimated to complete the trial by 31 October 2022 in collaboration with Masonic Cancer Center, University of Minnesota, USA [5]. 

In addition to the above companies some other biopharmaceutical companies are also involved in encouraging personalized medicines by targeting immune cells are: AstraZeneca, Acepodia, Affimed, Avid Biotics, Bristol-Myers Squibb, Celgene, Cellular Therapeutics, Celularity, Crispr Therapeutics, Dragonfly Therapeutics, Effector Therapeutics, Fate Therapeutics Inc., Fortress Biotech Inc., Genentech, Glycostem Therapeutics, Green Cross Lab Cell Korea, Gamida Cell, GT Biopharma, ImmuneOncia therapeutics, Korea [127,128], Innate Pharma, ImmunityBio, Inc. (NCT03387085), Intima Bioscience, Inc. (NCT04426669 and NCT03538613), Juno Therapeutics Inc., Kyowa Hakko Kirin, Kiadis Pharma, Mentrik Biotech, Multimmune GmbH, NantKwest Inc., Nektar Therapeutics, Nkarta Therapeutics, NOXXON Pharma, Northwest Biotherapeutics, ONK therapeutics, Roche Glycart, Rubius Therapeutics, Sanofi, Senti Biosciences, SignalRX Pharmaceuticals Inc., Sorrento Therapeutics Inc., XNK Therapeutics in collaboration with Sanofi’s and NextGenNK competence center coordinated by Karolinska institute conducting (EudraCT No: 2010-0223330-83 phase-I/II and NCT04558853) clinical trial, and Ziopharm Oncology Inc. [83,139,142,143,175], (Table 1 and Table 3).

Box 1 **Immune cells targeting intracellular checkpoint ‘CISH/SOCS-1’ in improving ICB-efficacy.**

■ **NK-cells targeting intracellular checkpoint CISH:** ONK therapeutics, Ireland, estimated to conduct Phase-I clinical trial (ONKT102, ONKT103 and ONKT104) by 2021𠄲2022 for the treatment of haematological malignancies (multiple myeloma and AML) and solid tumors (ovarian, NSCLC and breast cancers) [135].
■ **TILs targeting intracellular checkpoint CISH:** Intima Bioscience, UK, estimated to conduct Phase-I/II clinical trial (NCT04426669, NCT03538613) by 2021𠄲2022 for the treatment of wide range of tumor types and gastrointestinal cancer [1,2,5].
■ **DCs targeting intracellular checkpoint SOCS-1:** Military medical sciences, China, conducted Phase-I clinical trial (NCT01956630) in 2018 for the treatment of RAL [7,182].

Box 2 **Important links targeting intracellular checkpoint ‘CISH’ in developing personalized medicine (accessed on: 28 July 2021).**

https://clinicaltrials.gov/ct2/show/NCT04426669

https://www.onktherapeutics.com/pipeline/

https://youtu.be/-ejnruT_yo4

https://acir.org/weekly-digests/2020/october/a-new-internal-t-cell-checkpoint-cish

https://crisprmedicinenews.com/news/crispr-cas9-knockout-of-a-novel-cancer-checkpoint-unleashes-t-cell-reactivity-against-solid-tumours/

https://www.onktherapeutics.com/onk-therapeutics-secures-exclusive-global-license-to-patent-for-cish-knockout-in-nk-cells-for-the-treatment-of-cancer-from-australias-wehi/

https://www.evaluate.com/node/13152/pdf

https://www.nkartatx.com/news/06-22-20/

## 7. Conclusions

In this review, we have summarized the strategies to improve the efficacy of immune checkpoint blockade therapy by combining personalized medicines. In our opinion, we have put forwarded the strategies that are worth-considering regarding the importance of epitranscriptomics, in improving the efficacy of ICB-therapy. Moreover, combining immunotherapy by targeting intracellular immune checkpoints ‘CISH/SOCS-1’ in NK-cells, TILs and DCs would further potentiates the efficacy of ICB-therapy. We anticipate our investigation would boost clinicians and researchers in further strengthening the efficacy of ICB-antibodies by considering the significance of personalized medicines towards solving the issues largely associated with high-dose antibody toxicity and drug-resistance. Further investigation is warranted targeting CISH in DCs to check its immunotherapeutic competency in controlling Th1/Th2-associated immune disorders.

## 8. Future Prospective

In addition to NK-cells and T-cells; clinical trial co-targeting CISH-expressing DCs in combination with ICB-antibodies has not been scheduled yet providing an opportunity into exploring the unattended avenues of CISH as a new intracellular checkpoint. Several evidences (epitranscriptomics [202,203,204], microRNAs or CRISPR therapeutics [184,191]) certainly signpost the potential of targeting CISH-expressing DCs not only to overcome the issues associated with high-dose antibody toxicities and drug-resistances but also holds the potential to improve body‘s own defence mechanism by enhancing cellular immunity [14,16] as well as humoral immunities [205].

## Figures and Tables

**Table 1:** Epigenetic modifiers and microRNAs in improving the efficacy of ICB-therapy.

RNA (m6A)-Modifiers (Editors/Erasers/Effectors)					
RNA Modifiers	Disease Condition	Target	Disease Mechanism	Therapeutic Strategies	Ref.
**Writers** Mettl3/14	up-regulated in colorectal cancer and melanoma	IFNγ, STAT1, IRF1, Cxcl-9 and Cxcl-10	By reducing CD8^+^T-cells infiltrations in TME	CRISPR/cas9 silencing of Mettl3/14 via YTHDF2	[8]
Mettl-3	down-regulated in M1/M2-med. lung metastasis	Spred-2	By recruiting immunosuppresive T-reg and MDSCs	Overexpressing Mettl3 via polarizing M1/M2-macrophages	[9]
m^6^A	m^6^A-mediated regulation of PD-L1 in HNSCC	G2M checkpoint and PI3K/AKT/ mTOR signaling	Analysed via cancer genome atlas TCGA and GSE65858 cohort	By targeting m^6^A regulatated signature genes	[10]
**Erasers** FTO	up-regulated in melanoma	PD-1, CXCR4 and SOX10	Impairs anti-PD1 effect by reducing target gene expressions	Selective inhibition of FTO to enhance anti-PD1 effects	[11]
FTO	up-regulated in colon cancer	PD-L1	Up-regulates PD-L1 expression in IFNγ signaling-independent manner	Selective inhibition of FTO inhibits PD-L1 to control colon cancer	[12]
ALKBH5	up-regulated in melanoma	Mct4/Slc16a3	By recruiting immunosuppresive T-reg and MDSCs	Anti-ALKBH5 enhances the effect of anti-PD1 therapy.	[13]
**Readers** YTHDF1	up-regulated in solid tumors	Lysosomal cathepsins	Degrade neo-antigen and impair dendritic cell presentation	Anti-YTHDF1 suppress cathepsins and enhance DC cross-presentation	[14]
YTHDF2	up-regulated in LGG (brain tumor) and several other immune cells	PD-1, CTLA4, TIM3	Impair immune checkpoint signalling	Anti-YTHDF2 in combination with immunecheckpoint immunotherapy	[15,16]
**DNA and Histone Modifiers in ICB-Therapeutics**					
**Epigenetic Regulators**	**Disease Condition**	**Target**	**Mechanism**	**Therapeutic Strategies**	**Ref.**
DNA methylation	down-ragulates CTLA4 in HNSCC	CTLA4, CD28, CD80/86, ICOS	DNA methylation affects HNSCC	Selective DNA (DNMTs) inhibitors	[17]
DNA methylation	down-regulates PD-L1 in melanoma	Interfron signalling	cpG DNA methylation regulate melanoma		[18]
DNA methylation	up-regulates PD-1 & CTLA4 in NSCLC	PD-1 (PDCD-1) CTLA4	Hypo-methylation increases PD-1, CTLA4 expression in NSCLC	Selective DNA (5hmC) inhibitors	[19]
DNA methylation	up-regulates PD-L1 & PD-L2 in HNSCC	PD-L1 (CD274) PD-L2 (PDCD1LG2)	Hypo-methylation increases PD-L1 & PD-L2 expression	Combining DNA inh. with Nivolumab and Pembrolizumab	[20]
DNA methylation	up-regulates PD-L1 in CRC	PD-L1 (CD274)	DNA-methylation control PD-L1 exp.	Selective DNA (TETs) inhibitors	[21]
HDAC	up-ragulates CTLA4 in B-cell associated function	CTLA4 and LAG3	Tcf1 regulate CTLA4 expression in T_FH_-cells	HDAC*i* control CTLA4-mediated B-cell help	[22]
HDAC6	up-regulates PD-L1 in melanoma	PD-L1 (CD274) STAT3	HDAC6 increase PD-L1 expression by recruiting STAT3	HDAC6-inhibitor decreases PD-L1 by de-activating STAT3	[23]
Active H3K4me3	up-regulates PD-L1 in breast cancer	EMT-induced PD-L1 expression	Active H3K4me3 modifications in Breast cancer	Selective histone inhi. enhance the efficacy of ICB-Abs	[24]
Active H3K4me3	up-regulates PD-L1 in pancreatic cancer	PD-L1 (CD274)	MLL1 catalyzed H3K4me3 to bind with PD-L1 promoter and increase its expression	MLL1 inhibitor in combination with anti-PD-L1,anti-PD-1 improves efficacy	[25]
Repressive H3K27me3	down-regulates PD-L1 in HCC	PD-L1, IRF1	EZH2 negatively regulate PD-L1 exp. by recruiting repressive H3K27me3 in HCC	Selective H3K27me3 inhibitor could enhance ICB efficacy	[26]
HDAC*i* (Belinostat)	up-regulates PD-L1 & CTLA4 in HCC	Increase IFN-γ & reduce T-reg populations	Belinostat treatment increase anti-tumor immunity against HCC	Combining belinostat enhances the efficacy of ICB therapy	[27]
SAHA	Increases CTLA4 and Foxp3 exp. cardiac transplant	Foxp3 CTLA4	SAHA increases suppressive function of T-reg to prolong allograft survival	SAHA (HDACi) couls be a promissing immunosuppressive agent with CNI drug	[28]
H3Ac	up-regulates PD-L1 in drug resistant cancer cell	H3Ac enhance PD-L1 exp.	drug resistant issues in cancer cells	HDAC*i* in combination with anti-PD-L1	[29]
**MicroRNAs in ICB-Therapeutics**					
**miRNAs**	**Disease Condition**	**Target**	**Mechanism**	**Therapeutic Strategies**	**Ref.**
miR-15a,b miR-16, miR-193a-3p	down-regulated in MPM	Direct target of PD-L1	miR-15a, miR-16 and miR-193a-3p (−)vely regulates PD-L1	Respective miRNA mimics combined ICB-therapeutics	[30]
miR-17-5p	down-regulated in melanoma	Directly binds 3′-UTR PD-L1	miR-17-5p (−)vely regulates PD-L1	miR-17-5p mimics with anti-PD-L1 Abs	[31]
miR-18a (miR-140, 142, 340, 383)	up-regulated in cervical cancer	PI3K/AKT, WNK2, SOX6, p53 PTEN, MEK	miR-18a (+)vely and miR-140, 142, 340, 383 (−)ly regulates PD-L1	Respective miRNA antagomiR & mimics with ICB-therapy	[32]
miR-20b-21-130b	up-regulated in colorectal cancer	PTEN, B7-H1 (PD-1)	miRs (+)vely regulates B7-H1 (PD-1) exp.	Respective miRNAs AntagomiRs in combination with ICB-therapeutics	[33]
miR-21 (CD4^+^T-cells)	up-regulated in arthritis and GC	PDCD4, Th17, STAT5, T-reg	miR-21 (−)vely regulates PDCD4, PD-1		[34,35]
miR-23a-3p	up-regulated in (MΦ) liver cancer	PTEN, AKT pathways	miR-23a-3p (+)vely regulates PD-L1 exp.	Anti-miR-23a-3p (antagomiR therapy) with anti-PD-L1 Abs	[36]
miR-25-93- 106b cluster	down-regulated in pancreatic cancer	CXCL12, PD-L1	miR-25-93- 106b^−/−^ mice increases PD-L1	miR-93, miR-106b mimics with BET inh.	[37]
miR-28	melanoma	PD-1	miR-28 (−)vely regulates PD-1	miR-28 mimics	[38]
miR-33a	down-regulated in Lung A. carcinoma	PD-L1,CTLA4, PD-1, CAND1	miR-33a (−)vely regulates PD-1/PD-L1	miR-33a mimics with combined ICB-Abs	[39]
miR-34a	down-regulated in AML, lymphoma	EBF-1 and 3′-UTR PD-L1	miR-34a (−)vely regulates PD-L1 exp.	ICB therapy combined miRNA	[40,41,42,43,44]
miR-138-5p	down-regulated in CRC	Target 3′-UTR PD-L1	miR-138-5p (−)vely regulatesPD-L1 exp.	miR-138-5p mimics combined ICB-Abs	[45]
miR-140	down-regulated in NSCLC	miR-140/ PD-L1/cyclinE pathways	miR-140 target 3′-UTR PD-L1 (−)vely regulates its exp.	miR-140 mimics with anti-PD-L1 therapy	[46]
miR-142-5p	down-regulated in pancreatic cancer	miR-142-5p target 3′-UTR PD-L1	miR-142-5p (−)vely regulates PD-L1 exp.	miR-142-5p mimics + anti-PD-L1 therapy	[47]
miR-145	down-regulated in ovarian carcinoma	Cisplatin cMYc (TcF)	miR-145 (−)vely regulates PD-L1 exp.	miR-145 mimic (restoration therapy) with anti-PD-L1 Abs	[48]
miR-146a	up-regulated in melanoma	STAT1-IFNγ axis	miR-146a (+)vely regulates PD-L1 exp.	miR-146a antagomiR with anti-PD-L1 Abs	[49]
miR-148a -3p	down-regulated in dMMR/MSI-H CRC	miR-148a-3p binds to 3′-UTR PD-L1	miR-148a-3p (−)vely regulates PD-L1 exp.	Respective miRNA mimics with anti-PD-L1 therapy	[50]
miR-155	up-regulated in B-cell lymphoma	AKT and ERK	miR-155 (+)vely regulates PD-L1 exp.	miR-155 antagomiR + PD-L1 antagonists	[51]
miR-191-5p	down-regulated in colon-adenocarcinoma	PD-L1	miR-191-5p (−)vely regulates PD-L1 exp.	miR-191-5p mimics	[52]
miR-195	down-regulated in PC and DLBCL	PD-L1	miR-191-5p (−)vely regulates PD-L1 exp.	miR-191 mimics	[53,54]
miR-197	down-regulated in NSCLC	CKS1B/STAT3 (Bcl-2, c-Myc, CyclinD1)	miR-197 (−)vely regulates PD-L1 exp.	miR-193 mimics (replacement therapy) + ICB-therapeutics	[55]
miR-200b, miR-152	down-regulated in gastric cancer (GC)	B7-H1 (PD-1)	miR-200b and miR-152 (−)vely regulates B7-H1	Respective miRNA mimics combined PD-L1 antagonists	[43,56,57]
miR-214	down-regulated in B-cell lymphoma (DLBCL)	miR-214 atrget 3′-UTR PD-L1	miR-214 (−)vely regulates PD-L1 exp.	miR-214 mimic in combination with anti-PD-L1 Abs	[58]
miR-217	down-regulated in laryngeal cancer	AEG-1 and PD-L1	miR-217 (−)vely regulates PD-L1 exp	miR-217 mimics with anti-PD-L1 therapy	[59]
miR-324-5p miR-338-5p	downregulated in *Mycobateria*-responsive hedgehog sign	PD-L1, SHH signaling	(−)vely regulate PD-L1	miRNA mimics	[60]
miR-340	down-regulated in Cervical cancer	PD-L1	miR-340 (−)vely regulates PD-L1 exp.	miR-340 mimics	[61]
miR-375	down-regulated in HNSCC	JAK2	Inhibits JAK2-STAT1 axis suppressing PD-L1 exp.	miR-375 mimics	[62]
miR-424 (322)	down-regulated in ovarian cancer	PD-1/PD-L1, CD80/CTLA4	miR-424 (322) (−)vely regulates PD-1/PD-L1, CD80/CTLA4 exp.	miR-424 (322) mimics (restoration therapy) + ICB-therapeutics	[63]
	up-regulated in Colon cancer	CD28, CD80 and CD86	up regulated miR-424 impairs anti-tumor immunity	modified tumor-secreted EVs with miR-424 knocked down	[64]
miR-497-5p	down-regulated in RCC (ccRCC)	Cell proliferation	miR-497-5p (−)vely regulates PD-L1 exp.	miR-497-5p mimic with anti-PD-L1 Abs	[65]
miR-513	cholangiocytes in response to *C. parvum* infection	B7-H1 (PD-1)	miR-513 (−)vely regulates PD-1 exp.	miR-513 mimics	[66]
miR-570	down-regulated in gastric cancer	B7-H1 (PD-1)	SNP (polymorphism) disrupts miR-570- B7-H1 interactions	Restoration therapy combined ICB-Abs	[43,67]
miR-873	down-regulated in breast cancer	PI3K/Akt, ERK1/2 pathways	miR-873 (−)vely regulates PD-L1 by binding to 3′-UTR	miR-873 mimics with PD-1/PD-L1 inhibitor	[68]
miR-3127-5p	up-regulated in NSCLC	pSTAT3	Upregulates PD-L1 by suppressing p-STAT3	Anti-miR-3127-5p (antagomiR therapy)	[69]
miR-3609	down-regulated in breast cancer	PD-L1	miR-3609 (−)vely regulates PD-L1 exp.	miR-3609 mimics	[70]
miR-4717	down-regulated in HBV	PD-1	miR-4717 (−)vely regulates PD-1 exp.	miR-4717 mimics	[71]

**Table 2:** Biopharmaceutical companies developing immune checkpoint blockade (ICB)-antibodies.

Immune Cell Targeted Antibodies (Anti-PD-1 Therapy)						
Company	Antibody FDA Approval	Brand/ Other Name	Combination	Disease	Clinical Trial	Ref.
Bristol-Meyers Squibb	Nivolumab (Human IgG4) 2014	Opdivo®, BMS-936558, MDX-1106 ONO-4538	LAG3 (BMS-986016), B7-H3 (Enoblituzumab), KIR (Lirilumab), 4-1BB (Urelumab), ICOS (JTX-2011), CD27 (Varlilumab), GM.CD40L (vaccine for lung NSCLC)	Broad range of tumor types and Lymphomas	NCT01968109 NCT02817633 NCT01714739 NCT02253992 NCT02904226 NCT02335918 NCT02466568 NCT01673867	[83,84]
Medimmune		MEDI0680 (AMP-514)	-		NCT02118337 Phase I	[85,86]
Regeneron/ Sanofi		REGN2810	-		Phase I/II NCT02383212 NCT02760498	
Novartis		PDR001	GITR (GWN323)		NCT02740270	[87]
Merck	Pembrolizumab (Humanized IgG4k) 2014	Keytruda® MK-3475, lambrolizumab	B7-H3 (Enoblituzumab), Multi-kinase inhibitor (Sunitinib)	Melanoma, Lung, NSCLC, HNC, cervical, thyroid cancer	NCT02475213 NCT02599779 NCT01295827	[83,88]
Cure Tech	Pidilizumab (Humanized IgG1k)	CT-011	Pidilizumab (formerly CT-011), anti-delta like-1 (DLL1), anti-PD-1	Malignant gliomas	Phase I/ II NCT01952769	
Sanofi	Cemiplimab 2018	Libtayo®		Cervical cancer CSCC	Phase III	[83]
**Immune Cell Targeted Antibodies (Anti-CTLA4 Therapy)**						
Medarex/ Bristol-Meyers Squibb	Ipilimumab (IgG1 isotype) 2011	Yervoy® (BMS-734016, MDX-010, MDX-101)	Nivolumab, Gemcitabine, Cisplatin	Melanoma, SCLC, Bladder, prostate cancer	NCT00527735 NCT01524991 NCT00323882	[83,89,90,91,92]
Pfizer/ AstraZeneca	Tremolimumab (IgG2 isotype) 2015	Orphan drug approval, CP-675, 206		Metastatic melanoma, Solid Tumor	Phase III NCT02527434 NCT03703297	[93,94,95,96,97]
**Tumor Cell/APC-Targeted Antibodies (Anti-PD-L1/L2 Therapy)**						
Roche/ Genentech	Anti-PD-L1 Atezolizumab (Humanized IgG1k), 2016	Tecentriq®, MPDL3280A, RG7446, RO5541267	CD27 (Varlilumab), VEGF inhibitors (Bevacizumab cediranib)	Ovarian, Urothelial, Lung Cancer, HNCLC	NCT02543645 NCT02659384	[83]
Merck, EMD, Serono/Pfizer	Avelumab 2017	Bavencio® MSB0010718C	Metastatic MCC	Urothelial, RCC, Merkel	NCT02603432	[83,98]
Medimmune/ AstraZeneca	Anti-PD-L1 Durvalumab (Human IgG1k), 2017	Imfinzi® MEDI4736	Osimertinib, Olaparib and Sunitinib	NSCLC, Solid Tumor, urothelial carcinoma	Reference [70] NCT02221960 NCT02484404	[99,100,101]
Bristol-Meyers Squibb	Anti-PD-L1 (Human IgG4)	BMS-936559 (MDX1105)	-	HIV-1, Sepsis, NSCLC	Phase I NCT02028403	[102,103,104]
Amplimmune/ Glaxo Smith Klein	Anti-PD-L2	AMP-224	-	MCC	NCT02298946	[105]
	Anti-PD-L2 AMP-514 (fusion protein)	MEDI0680	-	kidney cancer, melanoma	Phase I NCT02013804	[86]

**Table 3:** Biotech companies/Universities entering into personalized medicine targeting intracellular immune checkpoints in combination with ICB-therapeutics.

Biopharmaceutical Company/University	Target	Combined Therapeutic Approach	Clinical Trial	Indication	Ref.
Natural Killer Cells (NK-cells) Clinicaltrials.gov, accessed on 15 July 2021					
ONK therapeutics (Ireland) 2015 www.onktherapeutics.com	CISH^−/−^ NK-cells NK-cells	**CISH^−/−^ NK-cells** in combination with ICB-antibodies	ONK102 ONK103 ONK104	M. Myeloma NSCLC AML	[135]
Fate Therapeutics San Diego, USA	iPSC-derived NK Cells (FT500)	Nivolumab (anti-PD-1) Pembrolizumab (anti-PD-1) Atezolizumab (anti-PD-L1) Interleukin-2 (IL-2)	NCT03841110 NCT04106167 (Phase-I)	Advanced solid tumors and lymphoma	[136,137,138,139,140]
Innate Pharma S. A	NK cell (NKG2A)	Durvalumab (Phase-I/II) Nivolumab (Phase-I) Ipilimumab (Phase-I) Nivolumab + 5-Aza (Ph-I)	NCT02671435 NCT01592370 NCT01750580 NCT02599649	Metastatic Cancer	[141,142]
Altor Biosciences corporation	IL-15 super agonist mediated NK-cells	Nivolumab (anti-PD-1)	NCT02523469 (Phase-I/II)	NSCLC	[142]
ImmunityBio, Inc.	High-affinity Natural Killer (haNK) Cell	Avelumab (Bavencio®) (anti-PD-L1)	NCT03387085 (Phase-I/II)	Triple Negative Breast Cancer	-
SignalRX Pharmaceuticals, Inc.	SF1126 (*dual inhibitor of PI3K and BRD4*)	Nivolumab (anti-PD-1)	NCT03059147	Advanced HCC	[83]
Effector Therapeutics	*Tomivosertib* (eFT-508)	Pembrolizumab (anti-PD-1)	NCT03616834 Phase-II Completed 2021	Solid tumors and NSCLC	[83]
NantKwest Inc., and Chan Soon-Shiong Institute for Medicine, USA	CD16-targeted NK-cell (haNK^TM^) with N-803 (IL-15 superagonist)	Avelumab (Bavencio®) (anti-PD-L1)	NCT03853317 (Phase-II)	Merkel cell carcinoma	[139,143]
National Cancer Institute, Naples	NK-cells (Tregs and NKs)	Nivolumab (anti-PD-1)	NCT03891485	Renal cell carcinoma	[144]
Gachon University & Severance hospital, Republic of Korea	Allogeneic NK-Cells (SMT-NK)	Pembrolizumab (anti-PD-1) Keytruda	NCT03937895 (Phase-I/II)	Biliary tract cancer	[139]
Fox Chase Cancer Center, USA	NK-cells and T-cells	Pembrolizumab (anti-PD-1)	NCT02535247 (Phase-I/II)	Lymphoma	[144,145,146]
Jilin University Hospital, China	NK-cells	PD-1 Ab	NCT03958097 (Phase-II)	Non-small cell lung cancer	[139]
MD Anderson Cancer Center, USA	DF1001 (a new molecule targeting NK-cell activations)	Drug: DF1001 Pembrolizumab (anti-PD-1)	NCT04143711 (Phase-I/II)	Advanced Solid Tumors	[139,144]
**T-Cells:** Tumor-Infiltrating Lymphocytes (TILs)					
Intima Bioscience, Inc. with University of Minnesota	**CISH-**deleted Tumor-Infiltrating Lymphocytes (TIL)	CISH checkpoint-deleted TILs combined with Cyclophosphamide, Fludarabine, Aldesleukin and ICB-therapeutics	NCT04426669 (Phase-I/II)	Solid tumors & gastro-intestinal cancers	[1,147]
	**CISH^−/−^ T-cells** (TILs)		NCT03538613 (Phase-I/II)	Gastro-intestinal cancers	[2,5]
Hangzhou Cancer Hospital in collabration with Anhui Kedgene Biotechnology Co.,Ltd	PD-1 Knockout T-Cells	CRISPR/Cas9-deleted PD-1 in T-Cells with hydrocortisone	NCT03081715 (Phase-I) Completed, 2018	Advanced Esophageal Squamous Cell Carcinoma	[2,144]
Sichuan University in collabration with Chengdu MedGenCell	PD-1 Knockout T-Cells	CRISPR/Cas9-deleted PD-1 in T-Cells with Cyclophosphamide	NCT02793856 (Phase-I) Completed, 2020	Metastatic Non-small Cell Lung Cancer	[2,144,148]
Peking University and (Cell Biotech)	PD-1 Knockout Engineered T Cells	PD-1-KO-T-cells with IL-2 and Cyclophosphamide	NCT02863913 NCT02867345 NCT02867332 (Phase-I)	Bladder, Prostate and Renal Cell Carcinoma	[2,5]
University of Pennsylvania, with Tmunity Therapeutics	NY-ESO-1 redirected autologous T cells	TCR-deleted and PD-1-deleted T cells	NCT03399448	Myeloma, melanoma and several cancers	[2,5,149]
Nanjing University Medical School	PD-1 Knockout EBV-CTLs	PD-1-KO-EBV-CTL with IL-2, Fludarabine and Cyclophosphamide	NCT03044743 (Phase-I/II)	EBV associated Malignancies	[2,5]
**Dendritic Cells** (DCs)					
H. Lee Moffitt Cancer Center, BMS and MultiVir, Inc.	DC-based p53 Vaccine	Ipilimumab (anti-CTLA4) Nivolumab (anti-PD-1)	NCT03406715 (Phase-II)	Small Cell Lung Cancer	[137]
Allife Medical Sc. and Technology Co., Ltd.	DC-NK YNYY-01 (DC-NK Cells)	Pembrolizumab (anti-PD-1) Keytruda	NCT03815084 (Phase-I)	Solid tumors	[144,150]
Bristol-Myers Squibb and Duke Cancer Inst.	DC Vaccines	Nivolumab (anti-PD-1)	NCT02529072 NCT02775292 (Phase-I)	Recurrent Brain Tumors	
Northwest Biotherapeutics, BMS and JCCC	Autologous DCs pulsed with tumor lysate	Nivolumab (anti-PD-1)	NCT03014804 (Phase-II)	Recurrent Glioblastoma	
University of Pennsylvania	Autologous DC pulsed peptide	Pembrolizumab (anti-PD-1)	NCT03092453 (Phase-I)	Advanced Melanoma	
Mayo Clinic in collabration with National Cancer Inst.	Autologous DC pulsed tumor Ags	Pembrolizumab (anti-PD-1)	NCT03035331 (Phase-I/II)	Aggressive Non-Hodgkin Lymphoma	
Oslo University Hospital in collabration with NCS and MSDC	Autologous DC	Pembrolizumab (anti-PD-1) Rituximab, GM-CSF and anti-TNF-alpha therapy	NCT02677155 (Phase-II)	Follicular Lymphoma	
Capital Medical Univ. in collabration with Duke Univ.	Autologous DC-CIK cell	Pembrolizumab Anti-PD-1 + DC-CIK (Ph-I) Anti-PD-1 alone (Ph-II)	NCT03190811 NCT03360630	Advanced Solid Tumors and NSCLC	
Sun Yat-sen University	DC-CIK cell (Cytokine-induced Killer Cell)	Anti-PD-1 antibody	NCT02886897 (Phase-I) Completed, 2019	Refractory Solid Tumors	
Beth Israel Deaconess Medical Center	Dendritic Cell Fusion Vaccine	Pidilizumab (anti-PD-1)	NCT01067287 (Phase-I)	Multiple Myeloma	
Cancer Insight in collabration with Elios Therapeutics, LLC	Autologous DC (TLPLDC Vaccine)	Checkpoint Inhibitor	NCT02678741 (Phase-I/II)	Metastatic Melanoma	
Grupo Espanol Multidisciplinario del Cancer Digestivo	Autologous DC Vaccine (AVEVAC)	Avelumab (Bavencio®) (anti-PD-L1)	NCT03152565 (Phase-I/II) Completed, 2020	Metastatic Colorectal Carcinoma	
Dana-Farber Cancer Institute in collabration with Celgene	DC/AML Fusion Vaccine	Durvalumab (Imfinzi®) (anti-PD-L1)	NCT03059485 (Phase-II)	Acute Myelogenous Leukemia	
Radboud University in collabration with Dutch Cancer Society	MiHA-loaded PD-L1/L2 silenced DC Vaccination	PD-L1/L2-silenced DC (siRNA silenced)	NCT02528682 (Phase-I/II) Completed, 2021	Hematological Malignancies	
Johns Hopkins University, USA	TLR3 agonist enhace DC activation	Anti-PD-1 in combination with DCs	-	Glioblastoma	[16]

## Data Availability

Not applicable.

## Abbreviations

AIA	Ag-induced arthritis
ALKBH5	alpha-ketoglutarate-dependent hydroxylase
CAND1	Cullin-associated NEDD8-dissociated protein 1
EZH2	Enhancer of zeste 2 polycomb repressive complex-2 subunit
EBF-1	Early B-cell factor-1
FTO	Fat Mass and Obesity Associated Protein
MPM	Malignant pleural mesothelioma
Spred2	Sprouty related EVH1 domain containing protein-2

## References

[1] Palmer D., Webber B., Patel Y., Johnson M., Kariya C., Lahr W., Parkhurst M., Gartner J., Prickett T., Lowery F. (2020). 333 Targeting the apical intracellular checkpoint CISH unleashes T cell neoantigen reactivity and effector program. J. Immunol. Ther. Cancer.

[2] Plieth J. Crispr: Nice Valuation, but Where’s the Clinical Trial?. https://www.evaluate.com/node/13152/pdf.

[3] Delconte R.B., Kolesnik T.B., Dagley L.F., Rautela J., Shi W., Putz E.M., Stannard K., Zhang J.G., Teh C., Firth M. (2016). CIS is a potent checkpoint in NK cell-mediated tumor immunity. Nat. Immunol..

[4] Putz E.M., Guillerey C., Kos K., Stannard K., Miles K., Delconte R.B., Takeda K., Nicholson S.E., Huntington N.D., Smyth M.J. (2017). Targeting cytokine signaling checkpoint CIS activates NK cells to protect from tumor initiation and metastasis. Oncoimmunology.

[5] Palmer D.C., Webber B.R., Patel Y., Johnson M.J., Kariya C.M., Lahr W.S., Parkhurst M.R., Gartner J.J., Prickett T.D., Lowery F.J. (2020). Internal checkpoint regulates T cellneoantigen reactivity and susceptibility to PD1 blockade. bioRxiv.

[6] Palmer D.C., Guittard G.C., Franco Z., Crompton J.G., Eil R.L., Patel S.J., Ji Y., Van Panhuys N., Klebanoff C.A., Sukumar M. (2015). Cish actively silences TCR signaling in CD8+ T cells to maintain tumor tolerance. J. Exp. Med..

[7] Wang D., Huang X.F., Hong B., Song X.T., Hu L., Jiang M., Zhang B., Ning H., Li Y., Xu C. (2018). Efficacy of intracellular immune checkpoint-silenced DC vaccine. JCI Insight.

[8] Wang L., Hui H., Agrawal K., Kang Y., Li N., Tang R., Yuan J., Rana T.M. (2020). m(6) A RNA methyltransferases METTL3/14 regulate immune responses to anti-PD-1 therapy. EMBO J..

[9] Yin H., Zhang X., Yang P., Zhang X., Peng Y., Li D., Yu Y., Wu Y., Wang Y., Zhang J. (2021). RNA m6A methylation orchestrates cancer growth and metastasis via macrophage reprogramming. Nat. Commun..

[10] Yi L., Wu G., Guo L., Zou X., Huang P. (2020). Comprehensive Analysis of the PD-L1 and Immune Infiltrates of m(6)A RNA Methylation Regulators in Head and Neck Squamous Cell Carcinoma. Mol. Ther. Nucleic Acids.

[11] Yang S., Wei J., Cui Y.H., Park G., Shah P., Deng Y., Aplin A.E., Lu Z., Hwang S., He C. (2019). m(6)A mRNA demethylase FTO regulates melanoma tumorigenicity and response to anti-PD-1 blockade. Nat. Commun..

[12] Tsuruta N., Tsuchihashi K., Ohmura H., Yamaguchi K., Ito M., Ariyama H., Kusaba H., Akashi K., Baba E. (2020). RNA N6-methyladenosine demethylase FTO regulates PD-L1 expression in colon cancer cells. Biochem. Biophys. Res. Commun..

[13] Li N., Kang Y., Wang L., Huff S., Tang R., Hui H., Agrawal K., Gonzalez G.M., Wang Y., Patel S.P. (2020). ALKBH5 regulates anti-PD-1 therapy response by modulating lactate and suppressive immune cell accumulation in tumor microenvironment. Proc. Natl. Acad. Sci. USA.

[14] Han D., Liu J., Chen C., Dong L., Liu Y., Chang R., Huang X., Liu Y., Wang J., Dougherty U. (2019). Anti-tumour immunity controlled through mRNA m(6)A methylation and YTHDF1 in dendritic cells. Nature.

[15] Lin X., Wang Z., Yang G., Wen G., Zhang H. (2020). YTHDF2 correlates with tumor immune infiltrates in lower-grade glioma. Aging (Albany NY).

[16] Garzon-Muvdi T., Theodros D., Luksik A.S., Maxwell R., Kim E., Jackson C.M., Belcaid Z., Ganguly S., Tyler B., Brem H. (2018). Dendritic cell activation enhances anti-PD-1 mediated immunotherapy against glioblastoma. Oncotarget.

[17] de Vos L., Grunwald I., Bawden E.G., Dietrich J., Scheckenbach K., Wiek C., Zarbl R., Bootz F., Landsberg J., Dietrich D. (2020). The landscape of CD28, CD80, CD86, CTLA4, and ICOS DNA methylation in head and neck squamous cell carcinomas. Epigenetics.

[18] Micevic G., Thakral D., McGeary M., Bosenberg M.W. (2019). PD-L1 methylation regulates PD-L1 expression and is associated with melanoma survival. Pigment Cell Melanoma Res..

[19] Marwitz S., Scheufele S., Perner S., Reck M., Ammerpohl O., Goldmann T. (2017). Epigenetic modifications of the immune-checkpoint genes CTLA4 and PDCD1 in non-small cell lung cancer results in increased expression. Clin. Epigenet..

[20] Franzen A., Vogt T.J., Muller T., Dietrich J., Schrock A., Golletz C., Brossart P., Bootz F., Landsberg J., Kristiansen G. (2018). PD-L1 (CD274) and PD-L2 (PDCD1LG2) promoter methylation is associated with HPV infection and transcriptional repression in head and neck squamous cell carcinomas. Oncotarget.

[21] Goltz D., Gevensleben H., Dietrich J., Dietrich D. (2017). PD-L1 (CD274) promoter methylation predicts survival in colorectal cancer patients. Oncoimmunology.

[22] Li F., Zhao X., Zhang Y., Shao P., Ma X., Paradee W.J., Liu C., Wang J., Xue H.H. (2021). TFH cells depend on Tcf1-intrinsic HDAC activity to suppress CTLA4 and guard B-cell help function. Proc. Natl. Acad. Sci. USA.

[23] Lienlaf M., Perez-Villarroel P., Knox T., Pabon M., Sahakian E., Powers J., Woan K.V., Lee C., Cheng F., Deng S. (2016). Essential role of HDAC6 in the regulation of PD-L1 in melanoma. Mol. Oncol..

[24] Darvin P., Sasidharan Nair V., Elkord E. (2019). PD-L1 Expression in Human Breast Cancer Stem Cells Is Epigenetically Regulated through Posttranslational Histone Modifications. J. Oncol..

[25] Lu C., Paschall A.V., Shi H., Savage N., Waller J.L., Sabbatini M.E., Oberlies N.H., Pearce C., Liu K. (2017). The MLL1-H3K4me3 Axis-Mediated PD-L1 Expression and Pancreatic Cancer Immune Evasion. J. Natl. Cancer Inst..

[26] Xiao G., Jin L.L., Liu C.Q., Wang Y.C., Meng Y.M., Zhou Z.G., Chen J., Yu X.J., Zhang Y.J., Xu J. (2019). EZH2 negatively regulates PD-L1 expression in hepatocellular carcinoma. J. Immunother. Cancer.

[27] Llopiz D., Ruiz M., Villanueva L., Iglesias T., Silva L., Egea J., Lasarte J.J., Pivette P., Trochon-Joseph V., Vasseur B. (2019). Enhanced anti-tumor efficacy of checkpoint inhibitors in combination with the histone deacetylase inhibitor Belinostat in a murine hepatocellular carcinoma model. Cancer Immunol. Immunother..

[28] Zhang X., Han S., Kang Y., Guo M., Hong S., Liu F., Fu S., Wang L., Wang Q.X. (2012). SAHA, an HDAC inhibitor, synergizes with tacrolimus to prevent murine cardiac allograft rejection. Cell. Mol. Immunol..

[29] Wang H., Fu C., Du J., Wang H., He R., Yin X., Li H., Li X., Wang H., Li K. (2020). Enhanced histone H3 acetylation of the PD-L1 promoter via the COP1/c-Jun/HDAC3 axis is required for PD-L1 expression in drug-resistant cancer cells. J. Exp. Clin. Cancer Res..

[30] Kao S.C., Cheng Y.Y., Williams M., Kirschner M.B., Madore J., Lum T., Sarun K.H., Linton A., McCaughan B., Klebe S. (2017). Tumor Suppressor microRNAs Contribute to the Regulation of PD-L1 Expression in Malignant Pleural Mesothelioma. J. Thorac. Oncol..

[31] Audrito V., Serra S., Stingi A., Orso F., Gaudino F., Bologna C., Neri F., Garaffo G., Nassini R., Baroni G. (2017). PD-L1 up-regulation in melanoma increases disease aggressiveness and is mediated through miR-17-5p. Oncotarget.

[32] Dong P., Xiong Y., Yu J., Chen L., Tao T., Yi S., Hanley S.J.B., Yue J., Watari H., Sakuragi N. (2019). Correction: Control of PD-L1 expression by miR-140/142/340/383 and oncogenic activation of the OCT4-miR-18a pathway in cervical cancer. Oncogene.

[33] Zhu J., Chen L., Zou L., Yang P., Wu R., Mao Y., Zhou H., Li R., Wang K., Wang W. (2014). MiR-20b, -21, and -130b inhibit PTEN expression resulting in B7-H1 over-expression in advanced colorectal cancer. Hum. Immunol..

[34] Iliopoulos D., Kavousanaki M., Ioannou M., Boumpas D., Verginis P. (2011). The negative costimulatory molecule PD-1 modulates the balance between immunity and tolerance via miR-21. Eur. J. Immunol..

[35] Zheng X., Dong L., Wang K., Zou H., Zhao S., Wang Y., Wang G. (2019). MiR-21 Participates in the PD-1/PD-L1 Pathway-Mediated Imbalance of Th17/Treg Cells in Patients After Gastric Cancer Resection. Ann. Surg. Oncol..

[36] Liu J., Fan L., Yu H., Zhang J., He Y., Feng D., Wang F., Li X., Liu Q., Li Y. (2019). Endoplasmic Reticulum Stress Causes Liver Cancer Cells to Release Exosomal miR-23a-3p and Up-regulate Programmed Death Ligand 1 Expression in Macrophages. Hepatology.

[37] Cioffi M., Trabulo S.M., Vallespinos M., Raj D., Kheir T.B., Lin M.L., Begum J., Baker A.M., Amgheib A., Saif J. (2017). The miR-25-93-106b cluster regulates tumor metastasis and immune evasion via modulation of CXCL12 and PD-L1. Oncotarget.

[38] Li Q., Johnston N., Zheng X., Wang H., Zhang X., Gao D., Min W. (2016). miR-28 modulates exhaustive differentiation of T cells through silencing programmed cell death-1 and regulating cytokine secretion. Oncotarget.

[39] Boldrini L., Giordano M., Niccoli C., Melfi F., Lucchi M., Mussi A., Fontanini G. (2017). Role of microRNA-33a in regulating the expression of PD-1 in lung adenocarcinoma. Cancer Cell. Int..

[40] Anastasiadou E., Stroopinsky D., Alimperti S., Jiao A.L., Pyzer A.R., Cippitelli C., Pepe G., Severa M., Rosenblatt J., Etna M.P. (2019). Epstein-Barr virus-encoded EBNA2 alters immune checkpoint PD-L1 expression by downregulating miR-34a in B-cell lymphomas. Leukemia.

[41] Kumar S., Ashraf M.U., Kumar A., Bae Y.S. (2021). Therapeutic Potential of microRNA Against Th2-associated Immune Disorders. Curr. Top. Med. Chem..

[42] Wang X., Li J., Dong K., Lin F., Long M., Ouyang Y., Wei J., Chen X., Weng Y., He T. (2015). Tumor suppressor miR-34a targets PD-L1 and functions as a potential immunotherapeutic target in acute myeloid leukemia. Cell. Signal..

[43] Boussiotis V.A. (2016). Molecular and Biochemical Aspects of the PD-1 Checkpoint Pathway. N. Engl. J. Med..

[44] Cortez M.A., Ivan C., Valdecanas D., Wang X., Peltier H.J., Ye Y., Araujo L., Carbone D.P., Shilo K., Giri D.K. (2016). PDL1 Regulation by p53 via miR-34. J. Natl. Cancer Inst..

[45] Zhao L., Yu H., Yi S., Peng X., Su P., Xiao Z., Liu R., Tang A., Li X., Liu F. (2016). The tumor suppressor miR-138-5p targets PD-L1 in colorectal cancer. Oncotarget.

[46] Xie W.B., Liang L.H., Wu K.G., Wang L.X., He X., Song C., Wang Y.Q., Li Y.H. (2018). MiR-140 Expression Regulates Cell Proliferation and Targets PD-L1 in NSCLC. Cell. Physiol. Biochem..

[47] Jia L., Xi Q., Wang H., Zhang Z., Liu H., Cheng Y., Guo X., Zhang J., Zhang Q., Zhang L. (2017). miR-142-5p regulates tumor cell PD-L1 expression and enhances anti-tumor immunity. Biochem. Biophys. Res. Commun..

[48] Sheng Q., Zhang Y., Wang Z., Ding J., Song Y., Zhao W. (2020). Cisplatin-mediated down-regulation of miR-145 contributes to up-regulation of PD-L1 via the c-Myc transcription factor in cisplatin-resistant ovarian carcinoma cells. Clin. Exp. Immunol..

[49] Mastroianni J., Stickel N., Andrlova H., Hanke K., Melchinger W., Duquesne S., Schmidt D., Falk M., Andrieux G., Pfeifer D. (2019). miR-146a Controls Immune Response in the Melanoma Microenvironment. Cancer Res..

[50] Ashizawa M., Okayama H., Ishigame T., Thar Min A.K., Saito K., Ujiie D., Murakami Y., Kikuchi T., Nakayama Y., Noda M. (2019). miRNA-148a-3p Regulates Immunosuppression in DNA Mismatch Repair-Deficient Colorectal Cancer by Targeting PD-L1. Mol. Cancer Res..

[51] Zheng Z., Sun R., Zhao H.J., Fu D., Zhong H.J., Weng X.Q., Qu B., Zhao Y., Wang L., Zhao W.L. (2019). MiR155 sensitized B-lymphoma cells to anti-PD-L1 antibody via PD-1/PD-L1-mediated lymphoma cell interaction with CD8+T cells. Mol. Cancer.

[52] Chen X.Y., Zhang J., Hou L.D., Zhang R., Chen W., Fan H.N., Huang Y.X., Liu H., Zhu J.S. (2018). Upregulation of PD-L1 predicts poor prognosis and is associated with miR-191-5p dysregulation in colon adenocarcinoma. Int. J. Immunopathol. Pharmacol..

[53] Tao Z., Xu S., Ruan H., Wang T., Song W., Qian L., Chen K. (2018). MiR-195/-16 Family Enhances Radiotherapy via T Cell Activation in the Tumor Microenvironment by Blocking the PD-L1 Immune Checkpoint. Cell. Physiol. Biochem..

[54] He B., Yan F., Wu C. (2018). Overexpressed miR-195 attenuated immune escape of diffuse large B-cell lymphoma by targeting PD-L1. Biomed. Pharmacother..

[55] Fujita Y., Yagishita S., Hagiwara K., Yoshioka Y., Kosaka N., Takeshita F., Fujiwara T., Tsuta K., Nokihara H., Tamura T. (2015). The clinical relevance of the miR-197/CKS1B/STAT3-mediated PD-L1 network in chemoresistant non-small-cell lung cancer. Mol. Ther..

[56] Chen L., Gibbons D.L., Goswami S., Cortez M.A., Ahn Y.H., Byers L.A., Zhang X., Yi X., Dwyer D., Lin W. (2014). Metastasis is regulated via microRNA-200/ZEB1 axis control of tumour cell PD-L1 expression and intratumoral immunosuppression. Nat. Commun..

[57] Xie G., Li W., Li R., Wu K., Zhao E., Zhang Y., Zhang P., Shi L., Wang D., Yin Y. (2017). Helicobacter Pylori Promote B7-H1 Expression by Suppressing miR-152 and miR-200b in Gastric Cancer Cells. PLoS ONE.

[58] Sun J.R., Zhang X., Zhang Y. (2019). MiR-214 prevents the progression of diffuse large B-cell lymphoma by targeting PD-L1. Cell Mol Biol Lett.

[59] Miao S., Mao X., Zhao S., Song K., Xiang C., Lv Y., Jiang H., Wang L., Li B., Yang X. (2017). miR-217 inhibits laryngeal cancer metastasis by repressing AEG-1 and PD-L1 expression. Oncotarget.

[60] Holla S., Stephen-Victor E., Prakhar P., Sharma M., Saha C., Udupa V., Kaveri S.V., Bayry J., Balaji K.N. (2016). Mycobacteria-responsive sonic hedgehog signaling mediates programmed death-ligand 1- and prostaglandin E2-induced regulatory T cell expansion. Sci. Rep..

[61] Dong P., Xiong Y., Yu J., Chen L., Tao T., Yi S., Hanley S.J.B., Yue J., Watari H., Sakuragi N. (2018). Control of PD-L1 expression by miR-140/142/340/383 and oncogenic activation of the OCT4-miR-18a pathway in cervical cancer. Oncogene.

[62] Wu Q., Zhao Y., Sun Y., Yan X., Wang P. (2018). miR-375 inhibits IFN-gamma-induced programmed death 1 ligand 1 surface expression in head and neck squamous cell carcinoma cells by blocking JAK2/STAT1 signaling. Oncol. Rep..

[63] Xu S., Tao Z., Hai B., Liang H., Shi Y., Wang T., Song W., Chen Y., OuYang J., Chen J. (2016). miR-424(322) reverses chemoresistance via T-cell immune response activation by blocking the PD-L1 immune checkpoint. Nat. Commun..

[64] Zhao X., Yuan C., Wangmo D., Subramanian S. (2021). Tumor-Secreted Extracellular Vesicles Regulate T-Cell Costimulation and Can Be Manipulated To Induce Tumor-Specific T-Cell Responses. Gastroenterology.

[65] Qu F., Ye J., Pan X., Wang J., Gan S., Chu C., Chu J., Zhang X., Liu M., He H. (2019). MicroRNA-497-5p down-regulation increases PD-L1 expression in clear cell renal cell carcinoma. J. Drug Target..

[66] Gong A.Y., Zhou R., Hu G., Liu J., Sosnowska D., Drescher K.M., Dong H., Chen X.M. (2010). Cryptosporidium parvum induces B7-H1 expression in cholangiocytes by down-regulating microRNA-513. J. Infect. Dis..

[67] Wang W., Li F., Mao Y., Zhou H., Sun J., Li R., Liu C., Chen W., Hua D., Zhang X. (2013). A miR-570 binding site polymorphism in the B7-H1 gene is associated with the risk of gastric adenocarcinoma. Hum. Genet..

[68] Gao L., Guo Q., Li X., Yang X., Ni H., Wang T., Zhao Q., Liu H., Xing Y., Xi T. (2019). MiR-873/PD-L1 axis regulates the stemness of breast cancer cells. EBioMedicine.

[69] Tang D., Zhao D., Wu Y., Yao R., Zhou L., Lu L., Gao W., Sun Y. (2018). The miR-3127-5p/p-STAT3 axis up-regulates PD-L1 inducing chemoresistance in non-small-cell lung cancer. J. Cell. Mol. Med..

[70] Li D., Wang X., Yang M., Kan Q., Duan Z. (2019). miR3609 sensitizes breast cancer cells to adriamycin by blocking the programmed death-ligand 1 immune checkpoint. Exp. Cell. Res..

[71] Zhang G., Li N., Li Z., Zhu Q., Li F., Yang C., Han Q., Lv Y., Zhou Z., Liu Z. (2015). microRNA-4717 differentially interacts with its polymorphic target in the PD1 3′ untranslated region: A mechanism for regulating PD-1 expression and function in HBV-associated liver diseases. Oncotarget.

[72] Wang D.Y., Johnson D.B., Davis E.J. (2018). Toxicities Associated With PD-1/PD-L1 Blockade. Cancer J..

[73] Jenkins R.W., Barbie D.A., Flaherty K.T. (2018). Mechanisms of resistance to immune checkpoint inhibitors. Br. J. Cancer.

[74] Fares C.M., Van Allen E.M., Drake C.G., Allison J.P., Hu-Lieskovan S. (2019). Mechanisms of Resistance to Immune Checkpoint Blockade: Why Does Checkpoint Inhibitor Immunotherapy Not Work for All Patients?. Am. Soc. Clin. Oncol. Educ. Book.

[75] Kalbasi A., Ribas A. (2020). Tumour-intrinsic resistance to immune checkpoint blockade. Nat. Rev. Immunol..

[76] Ishida Y., Agata Y., Shibahara K., Honjo T. (1992). Induced expression of PD-1, a novel member of the immunoglobulin gene superfamily, upon programmed cell death. EMBO J..

[77] Bashyam H. (2007). CTLA-4: From conflict to clinic. J. Exp. Med..

[78] Waterhouse P., Penninger J.M., Timms E., Wakeham A., Shahinian A., Lee K.P., Thompson C.B., Griesser H., Mak T.W. (1995). Lymphoproliferative disorders with early lethality in mice deficient in Ctla-4. Science.

[79] Tivol E.A., Borriello F., Schweitzer A.N., Lynch W.P., Bluestone J.A., Sharpe A.H. (1995). Loss of CTLA-4 leads to massive lymphoproliferation and fatal multiorgan tissue destruction, revealing a critical negative regulatory role of CTLA-4. Immunity.

[80] Dong H., Zhu G., Tamada K., Chen L. (1999). B7-H1, a third member of the B7 family, co-stimulates T-cell proliferation and interleukin-10 secretion. Nat. Med..

[81] Latchman Y., Wood C.R., Chernova T., Chaudhary D., Borde M., Chernova I., Iwai Y., Long A.J., Brown J.A., Nunes R. (2001). PD-L2 is a second ligand for PD-1 and inhibits T cell activation. Nat. Immunol..

[82] De Sousa Linhares A., Battin C., Jutz S., Leitner J., Hafner C., Tobias J., Wiedermann U., Kundi M., Zlabinger G.J., Grabmeier-Pfistershammer K. (2019). Therapeutic PD-L1 antibodies are more effective than PD-1 antibodies in blocking PD-1/PD-L1 signaling. Sci. Rep..

[83] Lucibello G., Mograbi B., Milano G., Hofman P., Brest P. (2021). PD-L1 regulation revisited: Impact on immunotherapeutic strategies. Trends. Mol. Med..

[84] Borghaei H., Paz-Ares L., Horn L., Spigel D.R., Steins M., Ready N.E., Chow L.Q., Vokes E.E., Felip E., Holgado E. (2015). Nivolumab versus Docetaxel in Advanced Nonsquamous Non-Small-Cell Lung Cancer. N. Engl. J. Med..

[85] Wu Z., Man S., Sun R., Li Z., Wu Y., Zuo D. (2020). Recent advances and challenges of immune checkpoint inhibitors in immunotherapy of non-small cell lung cancer. Int. Immunopharmacol..

[86] Naing A., Infante J., Goel S., Burris H., Black C., Marshall S., Achour I., Barbee S., May R., Morehouse C. (2019). Anti-PD-1 monoclonal antibody MEDI0680 in a phase I study of patients with advanced solid malignancies. J. Immunother. Cancer.

[87] Naing A., Gainor J.F., Gelderblom H., Forde P.M., Butler M.O., Lin C.C., Sharma S., Ochoa de Olza M., Varga A., Taylor M. (2020). A first-in-human phase 1 dose escalation study of spartalizumab (PDR001), an anti-PD-1 antibody, in patients with advanced solid tumors. J. Immunother. Cancer.

[88] Garon E.B., Rizvi N.A., Hui R., Leighl N., Balmanoukian A.S., Eder J.P., Patnaik A., Aggarwal C., Gubens M., Horn L. (2015). Pembrolizumab for the treatment of non-small-cell lung cancer. N. Engl. J. Med..

[89] Galsky M.D., Wang H., Hahn N.M., Twardowski P., Pal S.K., Albany C., Fleming M.T., Starodub A., Hauke R.J., Yu M. (2018). Phase 2 Trial of Gemcitabine, Cisplatin, plus Ipilimumab in Patients with Metastatic Urothelial Cancer and Impact of DNA Damage Response Gene Mutations on Outcomes. Eur. Urol..

[90] Slovin S.F., Higano C.S., Hamid O., Tejwani S., Harzstark A., Alumkal J.J., Scher H.I., Chin K., Gagnier P., McHenry M.B. (2013). Ipilimumab alone or in combination with radiotherapy in metastatic castration-resistant prostate cancer: Results from an open-label, multicenter phase I/II study. Ann. Oncol..

[91] Iwama S., De Remigis A., Callahan M.K., Slovin S.F., Wolchok J.D., Caturegli P. (2014). Pituitary expression of CTLA-4 mediates hypophysitis secondary to administration of CTLA-4 blocking antibody. Sci. Transl. Med..

[92] Hodi F.S., O’Day S.J., McDermott D.F., Weber R.W., Sosman J.A., Haanen J.B., Gonzalez R., Robert C., Schadendorf D., Hassel J.C. (2010). Improved survival with ipilimumab in patients with metastatic melanoma. N. Engl. J. Med..

[93] Tomillero A., Moral M.A. (2008). Gateways to clinical trials. Methods Find Exp. Clin. Pharmacol..

[94] Poust J. (2008). Targeting metastatic melanoma. Am. J. Health Syst. Pharm..

[95] Reuben J.M., Lee B.N., Li C., Gomez-Navarro J., Bozon V.A., Parker C.A., Hernandez I.M., Gutierrez C., Lopez-Berestein G., Camacho L.H. (2006). Biologic and immunomodulatory events after CTLA-4 blockade with ticilimumab in patients with advanced malignant melanoma. Cancer.

[96] Senan S., Okamoto I., Lee G.W., Chen Y., Niho S., Mak G., Yao W., Shire N., Jiang H., Cho B.C. (2020). Design and Rationale for a Phase III, Randomized, Placebo-controlled Trial of Durvalumab With or Without Tremelimumab After Concurrent Chemoradiotherapy for Patients With Limited-stage Small-cell Lung Cancer: The ADRIATIC Study. Clin. Lung. Cancer.

[97] Ribas A., Kefford R., Marshall M.A., Punt C.J., Haanen J.B., Marmol M., Garbe C., Gogas H., Schachter J., Linette G. (2013). Phase III randomized clinical trial comparing tremelimumab with standard-of-care chemotherapy in patients with advanced melanoma. J. Clin. Oncol..

[98] Powles T., Park S.H., Voog E., Caserta C., Valderrama B.P., Gurney H., Kalofonos H., Radulovic S., Demey W., Ullen A. (2020). Avelumab Maintenance Therapy for Advanced or Metastatic Urothelial Carcinoma. N. Engl. J. Med..

[99] Paz-Ares L., Dvorkin M., Chen Y., Reinmuth N., Hotta K., Trukhin D., Statsenko G., Hochmair M.J., Ozguroglu M., Ji J.H. (2019). Durvalumab plus platinum-etoposide versus platinum-etoposide in first-line treatment of extensive-stage small-cell lung cancer (CASPIAN): A randomised, controlled, open-label, phase 3 trial. Lancet.

[100] Antonia S.J., Villegas A., Daniel D., Vicente D., Murakami S., Hui R., Yokoi T., Chiappori A., Lee K.H., de Wit M. (2017). Durvalumab after Chemoradiotherapy in Stage III Non-Small-Cell Lung Cancer. N. Engl. J. Med..

[101] Antonia S.J., Villegas A., Daniel D., Vicente D., Murakami S., Hui R., Kurata T., Chiappori A., Lee K.H., de Wit M. (2018). Overall Survival with Durvalumab after Chemoradiotherapy in Stage III NSCLC. N. Engl. J. Med..

[102] Gay C.L., Bosch R.J., Ritz J., Hataye J.M., Aga E., Tressler R.L., Mason S.W., Hwang C.K., Grasela D.M., Ray N. (2017). Clinical Trial of the Anti-PD-L1 Antibody BMS-936559 in HIV-1 Infected Participants on Suppressive Antiretroviral Therapy. J. Infect. Dis..

[103] Hotchkiss R.S., Colston E., Yende S., Angus D.C., Moldawer L.L., Crouser E.D., Martin G.S., Coopersmith C.M., Brakenridge S., Mayr F.B. (2019). Immune Checkpoint Inhibition in Sepsis: A Phase 1b Randomized, Placebo-Controlled, Single Ascending Dose Study of Antiprogrammed Cell Death-Ligand 1 Antibody (BMS-936559). Crit. Care Med..

[104] Corrales L., Scilla K., Caglevic C., Miller K., Oliveira J., Rolfo C. (2018). Immunotherapy in Lung Cancer: A New Age in Cancer Treatment. Adv. Exp. Med. Biol..

[105] Floudas C.S., Brar G., Mabry-Hrones D., Duffy A.G., Wood B., Levy E., Krishnasamy V., Fioravanti S., Bonilla C.M., Walker M. (2019). A Pilot Study of the PD-1 Targeting Agent AMP-224 Used With Low-Dose Cyclophosphamide and Stereotactic Body Radiation Therapy in Patients With Metastatic Colorectal Cancer. Clin. Colorectal. Cancer.

[106] Nishimura H., Okazaki T., Tanaka Y., Nakatani K., Hara M., Matsumori A., Sasayama S., Mizoguchi A., Hiai H., Minato N. (2001). Autoimmune dilated cardiomyopathy in PD-1 receptor-deficient mice. Science.

[107] Nishimura H., Nose M., Hiai H., Minato N., Honjo T. (1999). Development of lupus-like autoimmune diseases by disruption of the PD-1 gene encoding an ITIM motif-carrying immunoreceptor. Immunity.

[108] Okazaki T., Tanaka Y., Nishio R., Mitsuiye T., Mizoguchi A., Wang J., Ishida M., Hiai H., Matsumori A., Minato N. (2003). Autoantibodies against cardiac troponin I are responsible for dilated cardiomyopathy in PD-1-deficient mice. Nat. Med..

[109] Wang J., Okazaki I.M., Yoshida T., Chikuma S., Kato Y., Nakaki F., Hiai H., Honjo T., Okazaki T. (2010). PD-1 deficiency results in the development of fatal myocarditis in MRL mice. Int. Immunol..

[110] Wang J., Yoshida T., Nakaki F., Hiai H., Okazaki T., Honjo T. (2005). Establishment of NOD-Pdcd1-/- mice as an efficient animal model of type I diabetes. Proc. Natl. Acad. Sci. USA.

[111] Yoshida T., Jiang F., Honjo T., Okazaki T. (2008). PD-1 deficiency reveals various tissue-specific autoimmunity by H-2b and dose-dependent requirement of H-2g7 for diabetes in NOD mice. Proc. Natl. Acad. Sci. USA.

[112] Okazaki T., Otaka Y., Wang J., Hiai H., Takai T., Ravetch J.V., Honjo T. (2005). Hydronephrosis associated with antiurothelial and antinuclear autoantibodies in BALB/c-Fcgr2b-/-Pdcd1-/- mice. J. Exp. Med..

[113] Grosso J.F., Jure-Kunkel M.N. (2013). CTLA-4 blockade in tumor models: An overview of preclinical and translational research. Cancer Immunol..

[114] Leach D.R., Krummel M.F., Allison J.P. (1996). Enhancement of antitumor immunity by CTLA-4 blockade. Science.

[115] Hodi F.S., Mihm M.C., Soiffer R.J., Haluska F.G., Butler M., Seiden M.V., Davis T., Henry-Spires R., MacRae S., Willman A. (2003). Biologic activity of cytotoxic T lymphocyte-associated antigen 4 antibody blockade in previously vaccinated metastatic melanoma and ovarian carcinoma patients. Proc. Natl. Acad. Sci. USA.

[116] Phan G.Q., Yang J.C., Sherry R.M., Hwu P., Topalian S.L., Schwartzentruber D.J., Restifo N.P., Haworth L.R., Seipp C.A., Freezer L.J. (2003). Cancer regression and autoimmunity induced by cytotoxic T lymphocyte-associated antigen 4 blockade in patients with metastatic melanoma. Proc. Natl. Acad. Sci. USA.

[117] Sharma P., Allison J.P. (2015). The future of immune checkpoint therapy. Science.

[118] Rosenberg S.A., Restifo N.P. (2015). Adoptive cell transfer as personalized immunotherapy for human cancer. Science.

[119] Pardoll D.M. (2012). The blockade of immune checkpoints in cancer immunotherapy. Nat. Rev. Cancer.

[120] Iwai Y., Hamanishi J., Chamoto K., Honjo T. (2017). Cancer immunotherapies targeting the PD-1 signaling pathway. J. Biomed. Sci..

[121] Chiossone L., Dumas P.Y., Vienne M., Vivier E. (2018). Natural killer cells and other innate lymphoid cells in cancer. Nat. Rev. Immunol..

[122] Waldman A.D., Fritz J.M., Lenardo M.J. (2020). A guide to cancer immunotherapy: From T cell basic science to clinical practice. Nat. Rev. Immunol..

[123] Wei S.C., Duffy C.R., Allison J.P. (2018). Fundamental Mechanisms of Immune Checkpoint Blockade Therapy. Cancer Discov..

[124] Barrueto L., Caminero F., Cash L., Makris C., Lamichhane P., Deshmukh R.R. (2020). Resistance to Checkpoint Inhibition in Cancer Immunotherapy. Transl. Oncol..

[125] Kumar S., Nagpal R., Kumar A., Ashraf M.U., Bae Y.S. (2021). Immunotherapeutic Potential of m6A-Modifiers and MicroRNAs in Controlling Acute Myeloid Leukaemia. Biomedicines.

[126] Zhao B.S., Roundtree I.A., He C. (2017). Post-transcriptional gene regulation by mRNA modifications. Nat. Rev. Mol. Cell. Biol..

[127] Gordon S.R., Maute R.L., Dulken B.W., Hutter G., George B.M., McCracken M.N., Gupta R., Tsai J.M., Sinha R., Corey D. (2017). PD-1 expression by tumour-associated macrophages inhibits phagocytosis and tumour immunity. Nature.

[128] Cheong K.H. Novel Immunotherapies to Combine with PD-1/PD-L1 Treatment. https://media.nature.com/original/magazine-assets/d43747-020-00338-3/d43747-020-00338-3.pdf.

[129] Dovedi S.J., Elder M.J., Yang C., Sitnikova S.I., Irving L., Hansen A., Hair J., Jones D.C., Hasani S., Wang B. (2021). Design and Efficacy of a Monovalent Bispecific PD-1/CTLA4 Antibody That Enhances CTLA4 Blockade on PD-1(+) Activated T Cells. Cancer Discov..

[130] Kakimi K., Karasaki T., Matsushita H., Sugie T. (2017). Advances in personalized cancer immunotherapy. Breast Cancer.

[131] Sahin U., Tureci O. (2018). Personalized vaccines for cancer immunotherapy. Science.

[132] Zhang H., Dai Z., Wu W., Wang Z., Zhang N., Zhang L., Zeng W.J., Liu Z., Cheng Q. (2021). Regulatory mechanisms of immune checkpoints PD-L1 and CTLA-4 in cancer. J. Exp. Clin. Cancer Res..

[133] Han Y., Liu D., Li L. (2020). PD-1/PD-L1 pathway: Current researches in cancer. Am. J. Cancer Res..

[134] Catela Ivkovic T., Voss G., Cornella H., Ceder Y. (2017). microRNAs as cancer therapeutics: A step closer to clinical application. Cancer Lett..

[135] Nowers C. Maximizing Synergy and Mitigating Resistance: Novel Dual-Targeted Natural Killer Cell Therapies for Cancer. https://www.onktherapeutics.com/wp/wp-content/uploads/2021/03/Nature-Biotech-Dealmakers-ONK-Therapeutics-March-2021.pdf.

[136] Ryan B.S.M., Gaidarova S., Abujarour R., Clarke R., Stokely L., Rogers P., Ge M., Robinson M., Rezner B., Lee T.T. (2018). Abstract 3576: FT500, an off-the-shelf NK cell cancer immunotherapy derived from a master pluripotent cell line, enhances T-cell activation and recruitment to overcome checkpoint blockade resistance. Cancer Res. Immunol..

[137] Guo H., He Y., Chen P., Wang L., Li W., Chen B., Liu Y., Wang H., Zhao S., Zhou C. (2020). Combinational immunotherapy based on immune checkpoints inhibitors in small cell lung cancer: Is this the beginning to reverse the refractory situation?. J. Thorac. Dis..

[138] Wang F., Lau J.K.C., Yu J. (2021). The role of natural killer cell in gastrointestinal cancer: Killer or helper. Oncogene.

[139] Shin M.H., Kim J., Lim S.A., Kim J., Kim S.J., Lee K.M. (2020). NK Cell-Based Immunotherapies in Cancer. Immune Netw..

[140] Goodridge J.P., Mahmood S., Zhu H., Gaidarova S., Blum R., Bjordahl R., Cichocki F., Chu H.-y., Bonello G., Lee T. (2019). FT596: Translation of First-of-Kind Multi-Antigen Targeted Off-the-Shelf CAR-NK Cell with Engineered Persistence for the Treatment of B Cell Malignancies. Blood.

[141] Bjordahl R., Gaidarova S., Woan K., Cichocki F., Bonello G., Robinson M., Ruller C., Pribadi M., Dinella J., Fong L. (2019). FT538: Preclinical Development of an Off-the-Shelf Adoptive NK Cell Immunotherapy with Targeted Disruption of CD38 to Prevent Anti-CD38 Antibody-Mediated Fratricide and Enhance ADCC in Multiple Myeloma When Combined with Daratumumab. Blood.

[142] Veluchamy J.P., Kok N., van der Vliet H.J., Verheul H.M.W., de Gruijl T.D., Spanholtz J. (2017). The Rise of Allogeneic Natural Killer Cells As a Platform for Cancer Immunotherapy: Recent Innovations and Future Developments. Front. Immunol..

[143] Zhang C., Hu Y., Shi C. (2020). Targeting Natural Killer Cells for Tumor Immunotherapy. Front. Immunol..

[144] U.S. National Library of Medicine. https://clinicaltrials.gov/.

[145] Barta S.K., Zain J., MacFarlane A.W.t., Smith S.M., Ruan J., Fung H.C., Tan C.R., Yang Y., Alpaugh R.K., Dulaimi E. (2019). Phase II Study of the PD-1 Inhibitor Pembrolizumab for the Treatment of Relapsed or Refractory Mature T-cell Lymphoma. Clin. Lymphoma Myeloma Leuk.

[146] Barta S.K., Fowler N.H., Zain J., Ruan J., Smith S.M., Schuster S.J., Nasta S.D., Svoboda J., Gerson J.N., Landsburg D.J. (2019). Pembrolizumab and Copanlisib for the Treatment of Relapsed or Refractory Mature T-Cell Lymphomas. Blood.

[147] Periasamy S., Dhiman R., Barnes P.F., Paidipally P., Tvinnereim A., Bandaru A., Valluri V.L., Vankayalapati R. (2011). Programmed death 1 and cytokine inducible SH2-containing protein dependent expansion of regulatory T cells upon stimulation With Mycobacterium tuberculosis. J. Infect. Dis..

[148] Lu Y., Xue J., Deng T., Zhou X., Yu K., Deng L., Huang M., Yi X., Liang M., Wang Y. (2020). Safety and feasibility of CRISPR-edited T cells in patients with refractory non-small-cell lung cancer. Nat. Med..

[149] Stadtmauer E.A., Fraietta J.A., Davis M.M., Cohen A.D., Weber K.L., Lancaster E., Mangan P.A., Kulikovskaya I., Gupta M., Chen F. (2020). CRISPR-engineered T cells in patients with refractory cancer. Science.

[150] Versteven M., Van den Bergh J.M.J., Marcq E., Smits E.L.J., Van Tendeloo V.F.I., Hobo W., Lion E. (2018). Dendritic Cells and Programmed Death-1 Blockade: A Joint Venture to Combat Cancer. Front. Immunol..

[151] Wang T., Kong S., Tao M., Ju S. (2020). The potential role of RNA N6-methyladenosine in Cancer progression. Mol. Cancer.

[152] Li Y., Gu J., Xu F., Zhu Q., Chen Y., Ge D., Lu C. (2020). Molecular characterization, biological function, tumor microenvironment association and clinical significance of m6A regulators in lung adenocarcinoma. Brief. Bioinform..

[153] Zhang B., Wu Q., Li B., Wang D., Wang L., Zhou Y.L. (2020). m(6)A regulator-mediated methylation modification patterns and tumor microenvironment infiltration characterization in gastric cancer. Mol Cancer.

[154] Han S.H., Choe J. (2020). Diverse molecular functions of m(6)A mRNA modification in cancer. Exp. Mol. Med..

[155] Dai X.Y., Shi L., Li Z., Yang H.Y., Wei J.F., Ding Q. (2021). Main N6-Methyladenosine Readers: YTH Family Proteins in Cancers. Front. Oncol..

[156] Elcheva I.A., Spiegelman V.S. (2021). Targeting RNA-binding proteins in acute and chronic leukemia. Leukemia.

[157] Hou G., Zhao X., Li L., Yang Q., Liu X., Huang C., Lu R., Chen R., Wang Y., Jiang B. (2021). SUMOylation of YTHDF2 promotes mRNA degradation and cancer progression by increasing its binding affinity with m6A-modified mRNAs. Nucleic Acids Res..

[158] Wang X., Lu Z., Gomez A., Hon G.C., Yue Y., Han D., Fu Y., Parisien M., Dai Q., Jia G. (2014). N6-methyladenosine-dependent regulation of messenger RNA stability. Nature.

[159] Kim D.J., Iwasaki A. (2019). YTHDF1 Control of Dendritic Cell Cross-Priming as a Possible Target of Cancer Immunotherapy. Biochemistry.

[160] Kachroo N., Valencia T., Warren A.Y., Gnanapragasam V.J. (2013). Evidence for downregulation of the negative regulator SPRED2 in clinical prostate cancer. Br. J. Cancer.

[161] Barbieri I., Tzelepis K., Pandolfini L., Shi J., Millan-Zambrano G., Robson S.C., Aspris D., Migliori V., Bannister A.J., Han N. (2017). Promoter-bound METTL3 maintains myeloid leukaemia by m(6)A-dependent translation control. Nature.

[162] Gulati P., Cheung M.K., Antrobus R., Church C.D., Harding H.P., Tung Y.C., Rimmington D., Ma M., Ron D., Lehner P.J. (2013). Role for the obesity-related FTO gene in the cellular sensing of amino acids. Proc. Natl. Acad. Sci. USA.

[163] Singh B., Kinne H.E., Milligan R.D., Washburn L.J., Olsen M., Lucci A. (2016). Important Role of FTO in the Survival of Rare Panresistant Triple-Negative Inflammatory Breast Cancer Cells Facing a Severe Metabolic Challenge. PLoS ONE.

[164] Su R., Dong L., Li Y., Gao M., Han L., Wunderlich M., Deng X., Li H., Huang Y., Gao L. (2020). Targeting FTO Suppresses Cancer Stem Cell Maintenance and Immune Evasion. Cancer Cell.

[165] Fujimura T., Kambayashi Y., Aiba S. (2012). Crosstalk between regulatory T cells (Tregs) and myeloid derived suppressor cells (MDSCs) during melanoma growth. Oncoimmunology.

[166] Adhikari S., Xiao W., Zhao Y.L., Yang Y.G. (2016). m(6)A: Signaling for mRNA splicing. RNA Biol..

[167] Zhao X., Yang Y., Sun B.F., Shi Y., Yang X., Xiao W., Hao Y.J., Ping X.L., Chen Y.S., Wang W.J. (2014). FTO-dependent demethylation of N6-methyladenosine regulates mRNA splicing and is required for adipogenesis. Cell Res..

[168] Selberg S., Seli N., Kankuri E., Karelson M. (2021). Rational Design of Novel Anticancer Small-Molecule RNA m6A Demethylase ALKBH5 Inhibitors. ACS Omega.

[169] Ding Z., Li Q., Zhang R., Xie L., Shu Y., Gao S., Wang P., Su X., Qin Y., Wang Y. (2021). Personalized neoantigen pulsed dendritic cell vaccine for advanced lung cancer. Signal. Transduct. Target. Ther..

[170] Yoshimura A., Ito M., Chikuma S., Akanuma T., Nakatsukasa H. (2018). Negative Regulation of Cytokine Signaling in Immunity. Cold Spring Harb. Perspect. Biol..

[171] Yoshimura A., Nishinakamura H., Matsumura Y., Hanada T. (2005). Negative regulation of cytokine signaling and immune responses by SOCS proteins. Arthritis Res. Ther..

[172] Shouda T., Yoshida T., Hanada T., Wakioka T., Oishi M., Miyoshi K., Komiya S., Kosai K., Hanakawa Y., Hashimoto K. (2001). Induction of the cytokine signal regulator SOCS3/CIS3 as a therapeutic strategy for treating inflammatory arthritis. J. Clin. Investig..

[173] Hunter M.G., Jacob A., O’Donnell L.C., Agler A., Druhan L.J., Coggeshall K.M., Avalos B.R. (2004). Loss of SHIP and CIS recruitment to the granulocyte colony-stimulating factor receptor contribute to hyperproliferative responses in severe congenital neutropenia/acute myelogenous leukemia. J. Immunol..

[174] Ochoa D., Hercules A., Carmona M., Suveges D., Gonzalez-Uriarte A., Malangone C., Miranda A., Fumis L., Carvalho-Silva D., Spitzer M. (2021). Open Targets Platform: Supporting systematic drug-target identification and prioritisation. Nucleic Acids Res..

[175] Martz L. Innate harmony. https://www.innate-pharma.com/sites/default/files/072816in_coverstory_innateharmony.pdf.

[176] Trengove M.C., Ward A.C. (2013). SOCS proteins in development and disease. Am. J. Clin. Exp. Immunol..

[177] Hernandez C., Bogdanov P., Gomez-Guerrero C., Sampedro J., Sola-Adell C., Espejo C., Garcia-Ramirez M., Prieto I., Egido J., Simo R. (2019). SOCS1-Derived Peptide Administered by Eye Drops Prevents Retinal Neuroinflammation and Vascular Leakage in Experimental Diabetes. Int. J. Mol. Sci..

[178] Chikuma S., Kanamori M., Mise-Omata S., Yoshimura A. (2017). Suppressors of cytokine signaling: Potential immune checkpoint molecules for cancer immunotherapy. Cancer Sci..

[179] Bernard P.-L., Delconte R.B., Pastor S., Laletin V., Goubard A., Josselin E., Castellano R., Vernerey J., Vivier E., Huntington N.D. (2021). CISH targeting in NK cells activates natural cytotoxicity receptor signaling and reduce cell exhaustion to unsilence primary anti-tumor response. bioRxiv.

[180] Felices M., Lenvik A.J., McElmurry R., Chu S., Hinderlie P., Bendzick L., Geller M.A., Tolar J., Blazar B.R., Miller J.S. (2018). Continuous treatment with IL-15 exhausts human NK cells via a metabolic defect. JCI Insight.

[181] Andre P., Denis C., Soulas C., Bourbon-Caillet C., Lopez J., Arnoux T., Blery M., Bonnafous C., Gauthier L., Morel A. (2018). Anti-NKG2A mAb Is a Checkpoint Inhibitor that Promotes Anti-tumor Immunity by Unleashing Both T and NK Cells. Cell.

[182] Gilboa E. (2004). Knocking the SOCS1 off dendritic cells. Nat. Biotechnol..

[183] Shen L., Evel-Kabler K., Strube R., Chen S.Y. (2004). Silencing of SOCS1 enhances antigen presentation by dendritic cells and antigen-specific anti-tumor immunity. Nat. Biotechnol..

[184] Miah M.A., Yoon C.H., Kim J., Jang J., Seong Y.R., Bae Y.S. (2012). CISH is induced during DC development and regulates DC-mediated CTL activation. Eur. J. Immunol..

[185] Kobayashi T., Yoshimura A. (2005). Keeping DCs awake by putting SOCS1 to sleep. Trends Immunol..

[186] Zhang W., Song Z., Xiao J., Liu X., Luo Y., Yang Z., Luo R., Li A. (2019). Blocking the PD-1/PD-L1 axis in dendritic cell-stimulated Cytokine-Induced Killer Cells with pembrolizumab enhances their therapeutic effects against hepatocellular carcinoma. J. Cancer.

[187] Lim T.S., Chew V., Sieow J.L., Goh S., Yeong J.P., Soon A.L., Ricciardi-Castagnoli P. (2016). PD-1 expression on dendritic cells suppresses CD8(+) T cell function and antitumor immunity. Oncoimmunology.

[188] Peng Q., Qiu X., Zhang Z., Zhang S., Zhang Y., Liang Y., Guo J., Peng H., Chen M., Fu Y.X. (2020). PD-L1 on dendritic cells attenuates T cell activation and regulates response to immune checkpoint blockade. Nat. Commun..

[189] Go D.M., Lee S.H., Lee S.H., Woo S.H., Kim K., Kim K., Park K.S., Park J.H., Ha S.J., Kim W.H. (2021). Programmed Death Ligand 1-Expressing Classical Dendritic Cells MitigateHelicobacter-Induced Gastritis. Cell. Mol. Gastroenterol. Hepatol..

[190] Zhu H., Blum R.H., Bernareggi D., Ask E.H., Wu Z., Hoel H.J., Meng Z., Wu C., Guan K.L., Malmberg K.J. (2020). Metabolic Reprograming via Deletion of CISH in Human iPSC-Derived NK Cells Promotes In Vivo Persistence and Enhances Anti-tumor Activity. Cell Stem Cell.

[191] Li H.B., Tong J., Zhu S., Batista P.J., Duffy E.E., Zhao J., Bailis W., Cao G., Kroehling L., Chen Y. (2017). m(6)A mRNA methylation controls T cell homeostasis by targeting the IL-7/STAT5/SOCS pathways. Nature.

[192] Kumar S., Jeong Y., Ashraf M.U., Bae Y.S. (2019). Dendritic Cell-Mediated Th2 Immunity and Immune Disorders. Int. J. Mol. Sci..

[193] Huemer F., Leisch M., Geisberger R., Zaborsky N., Greil R. (2021). miRNA-Based Therapeutics in the Era of Immune-Checkpoint Inhibitors. Pharmaceuticals (Basel).

[194] Skafi N., Fayyad-Kazan M., Badran B. (2020). Immunomodulatory role for MicroRNAs: Regulation of PD-1/PD-L1 and CTLA-4 immune checkpoints expression. Gene.

[195] Kumar S., Kim Y. (2017). An endoparasitoid wasp influences host DNA methylation. Sci. Rep..

[196] Kumar S., Venkata P., Kim Y. (2016). Suppressive activity of a viral histone H4 against two host chromatin remodelling factors: Lysine demethylase and SWI/SNF. J. Gen. Virol..

[197] He C., Lan F. (2021). RNA m(6)A meets transposable elements and chromatin. Protein Cell.

[198] Sharma P., Allison J.P. (2015). Immune checkpoint targeting in cancer therapy: Toward combination strategies with curative potential. Cell.

[199] Hu Z., Ott P.A., Wu C.J. (2018). Towards personalized, tumour-specific, therapeutic vaccines for cancer. Nat. Rev. Immunol..

[200] Blass E., Ott P.A. (2021). Advances in the development of personalized neoantigen-based therapeutic cancer vaccines. Nat. Rev. Clin. Oncol..

[201] Perrier A., Didelot A., Laurent-Puig P., Blons H., Garinet S. (2020). Epigenetic Mechanisms of Resistance to Immune Checkpoint Inhibitors. Biomolecules.

[202] Wang H., Hu X., Huang M., Liu J., Gu Y., Ma L., Zhou Q., Cao X. (2019). Mettl3-mediated mRNA m(6)A methylation promotes dendritic cell activation. Nat. Commun..

[203] Wu H., Xu Z., Wang Z., Ren Z., Li L., Ruan Y. (2020). Exosomes from dendritic cells with Mettl3 gene knockdown prevent immune rejection in a mouse cardiac allograft model. Immunogenetics.

[204] Feng Y., Dong H., Sun B., Hu Y., Yang Y., Jia Y., Jia L., Zhong X., Zhao R. (2021). METTL3/METTL14 Transactivation and m(6)A-Dependent TGF-beta1 Translation in Activated Kupffer Cells. Cell Mol. Gastroenterol. Hepatol..

[205] Yao Y., Yang Y., Guo W., Xu L., You M., Zhang Y.C., Sun Z., Cui X., Yu G., Qi Z. (2021). METTL3-dependent m(6)A modification programs T follicular helper cell differentiation. Nat. Commun..

